# CO confers neuroprotection via activating the PERK-calcineurin pathway and inhibiting necroptosis

**DOI:** 10.1038/s41420-025-02530-9

**Published:** 2025-05-27

**Authors:** Jeongmin Park, LiHua Jin, Hyun-Chul Song, Yingqing Chen, Eun Young Jang, Gyu Hwan Park, Chae Ha Yang, Stefan W. Ryter, Jeong Woo Park, Min Zheng, Yeonsoo Joe, Hun Taeg Chung

**Affiliations:** 1 College of Korean Medicine, Daegu Haany University, Gyeongsan, Republic of Korea; 2 School of Nursing, YanBian University, Yanji, China; 3 School of Biological Sciences, University of Ulsan, Ulsan, Korea; 4 Department of Pharmacology, Dalian University Medical College, Dalian, China; 5 Center for Convergence Toxicology Research, Korea Institute of Toxicology, Daejeon, Korea; 6 College of Pharmacy, Research Institute of Pharmaceutical Sciences, Kyungpook National University, Daegu, Korea; 7 College of Korean Medicine, Daegu Haany University, Daegu, Korea; 8 Proterris Inc., Boston, MA USA; 9 Department of Neurology, Affiliated Hospital of YanBian University, Yanji, China

**Keywords:** Stress signalling, Cell death in the nervous system

## Abstract

Neurodegenerative diseases, such as Parkinson’s disease (PD) and Alzheimer’s disease (AD), are marked by progressive neuronal loss. Regulated cell death programs (i.e., necroptosis) as well as homeostatic mechanisms (i.e., autophagy) can modulate disease pathogenesis. Low-dose carbon monoxide (CO) has been shown to activate cytoprotective responses in various models of tissue injury. Our study investigates the protective roles of CO in neurodegenerative disease through the modulation of necroptosis and autophagy programs. We found that CO activates the Protein kinase RNA (PKR)-like ER kinase (PERK) branch of the unfolded protein response (UPR) and the calcineurin pathway, leading to significant neuroprotective effects in cellular and mouse models of PD. CO-induced PERK activation promotes the nuclear translocation of transcription factor EB (TFEB). Subsequently, TFEB enhances autophagy through increased expression of autophagy-related genes and inhibits necroptosis by suppressing the phosphorylation and oligomerization of Mixed Lineage Kinase Domain-Like Pseudokinase (MLKL), a key necroptosis regulator. Furthermore, CO enhances the expression of Beclin 1, which inhibits necroptosis, independently of its autophagic function, by regulating MLKL oligomerization. Our findings suggest that modulation of the PERK-calcineurin pathway and downstream activation of cellular defense mechanisms by CO may serve as a promising therapeutic approach to mitigate neuronal loss in neurodegenerative diseases.

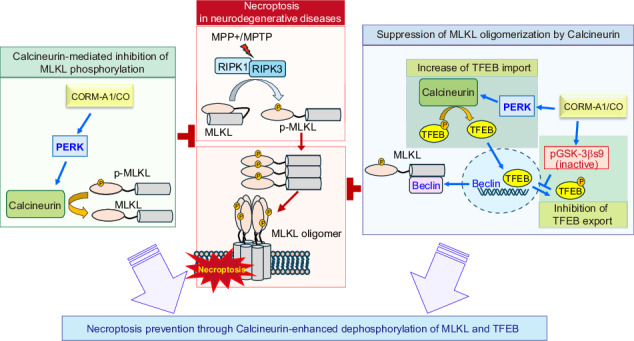

## Introduction

The growth of the elderly population has led to an increase in the prevalence of age-related neurodegenerative diseases [1, 2]. These diseases are characterized by several pathological features, including the accumulation of pathogenic proteins [3], dysfunction in synaptic and neuronal networks [4], aberrant proteostasis [5], cytoskeletal abnormalities [6], altered energy metabolism [7], DNA and RNA anomalies [8, 9], inflammation [9], and neuronal cell death [10, 11]. Notably, neuronal cell death, which can be exacerbated by age-related damage, is a natural and necessary process for maintaining tissue balance, preserving organ integrity, and ensuring proper neuronal function. Ongoing research into the mechanisms of neuronal cell death will provide essential insights into neurodegenerative diseases. The primary modes of neuronal cell death that have been identified include apoptosis, necroptosis, ferroptosis, pyroptosis, and mitochondrial permeability transition. Necroptosis, in particular, has been identified as playing a crucial role in the pathogenesis of several neurodegenerative diseases [12–15], such as Parkinson’s disease (PD) [16] and Alzheimer’s disease (AD) [17]. Necroptosis is regulated by a pathway in which Mixed Lineage Kinase Domain-Like Pseudokinase (MLKL) is phosphorylated by Receptor-Interacting Protein Kinase 3 (RIPK3), a downstream component of TNF-induced necroptosis. In turn, phosphorylated MLKL undergoes oligomerization, which mediates plasma membrane permeabilization [18, 19].

Autophagy is a cellular homeostatic mechanism for the turnover of cellular constituents, including organelles and proteins. Recent studies show that Beclin 1, an autophagy protein, can inhibit necroptosis by interacting with MLKL, which reduces MLKL oligomerization, in an autophagy-independent manner [20]. Further, Beclin 1 selectively inhibits MLKL oligomerization, but not MLKL phosphorylation. Additionally, the autophagy protein ATG16L1 was shown to prevent necroptosis in the intestinal epithelium, indicating a role for autophagy in protecting against necroptosis [21]. However, the molecular mechanisms by which autophagy proteins regulate necroptosis remain incompletely understood.

Transcriptional factor EB (TFEB), a major regulator of the autophagy-lysosomal system, can trigger the transcription of target genes responsible for the lysosomal biogenesis, in particular, Beclin 1 [22]. Thus, TFEB can mediate cellular autophagy by enhancing Beclin 1 transcription [23]. The regulation of TFEB is strictly governed by post-translational modifications and interactions with other proteins [20, 24]. When nutrients are abundant, TFEB is predominantly phosphorylated and inactive, residing in the cytosol due to the action of mTORC1 or ERK2 [25–27]. Conversely, during nutrient deprivation, TFEB will translocate to the nucleus, where it binds to its target genes, thereby initiating autophagy and enhancing lysosomal activities by promoting lysosomal Ca^2+^ release and activating calcineurin [28, 29]. Moreover, TFEB nuclear import is regulated by calcineurin-mediated phosphorylation, whereas its nuclear export is regulated by phosphorylation of S142 by mTORC1 and S138 by GSK-3β, in proximity of its nuclear export signal [30, 31]. The limitation of a nuclear import-export cycle of TFEB may regulate the enhancement of lysosomal biogenesis and autophagy [32–34]. Therefore, with the goal of augmenting autophagy activity for therapeutic effects on damaged cells, we sought to elucidate the mechanism(s) that regulate the nuclear import-export cycle of TFEB.

Carbon monoxide (CO) is an endogenously produced gas with hormetic effects. Prior studies have established that CO can modulate intracellular signaling pathways and provide anti-inflammatory effects in animal models of tissue injury [35–37]. CO can activate the PKR-like ER kinase (PERK), leading to an increase in TFEB nuclear import *via* Ca^2+^-calcineurin activation [36]. We have also demonstrated that CO inhibits GSK-3β activation in a dextran sulfate sodium-induced colitis model [38]. This suggests a potential mechanism for inhibiting necroptosis by preventing MLKL polymerization, which is initiated by increasing Beclin 1 levels *via* sustained TFEB nuclear import and reduced export. Our current studies suggest that CO activates this pathway to prevent necroptosis in neuronal cells. Additionally, our study is the first to demonstrate that CO-induced activation of the PERK-calcineurin pathway promotes MLKL dephosphorylation, thereby preventing necroptosis in neuronal cells. Our in vivo studies using a PD model induced by 1-Methyl-4-phenyl-1,2,3,6-tetrahydropyrine (MPTP) [39] revealed that the inhibition of necroptosis by CO significantly enhances neuronal cell survival and functionality. Thus, treatment with low concentration CO may represent an innovative therapeutic approach to protect against neurodegenerative diseases by preventing necroptosis in neuronal cells.

## Results

### CO-induced calcineurin activity suppresses necroptosis through MLKL dephosphorylation in a model of MPTP-induced neuronal injury

**Fig. 1:**
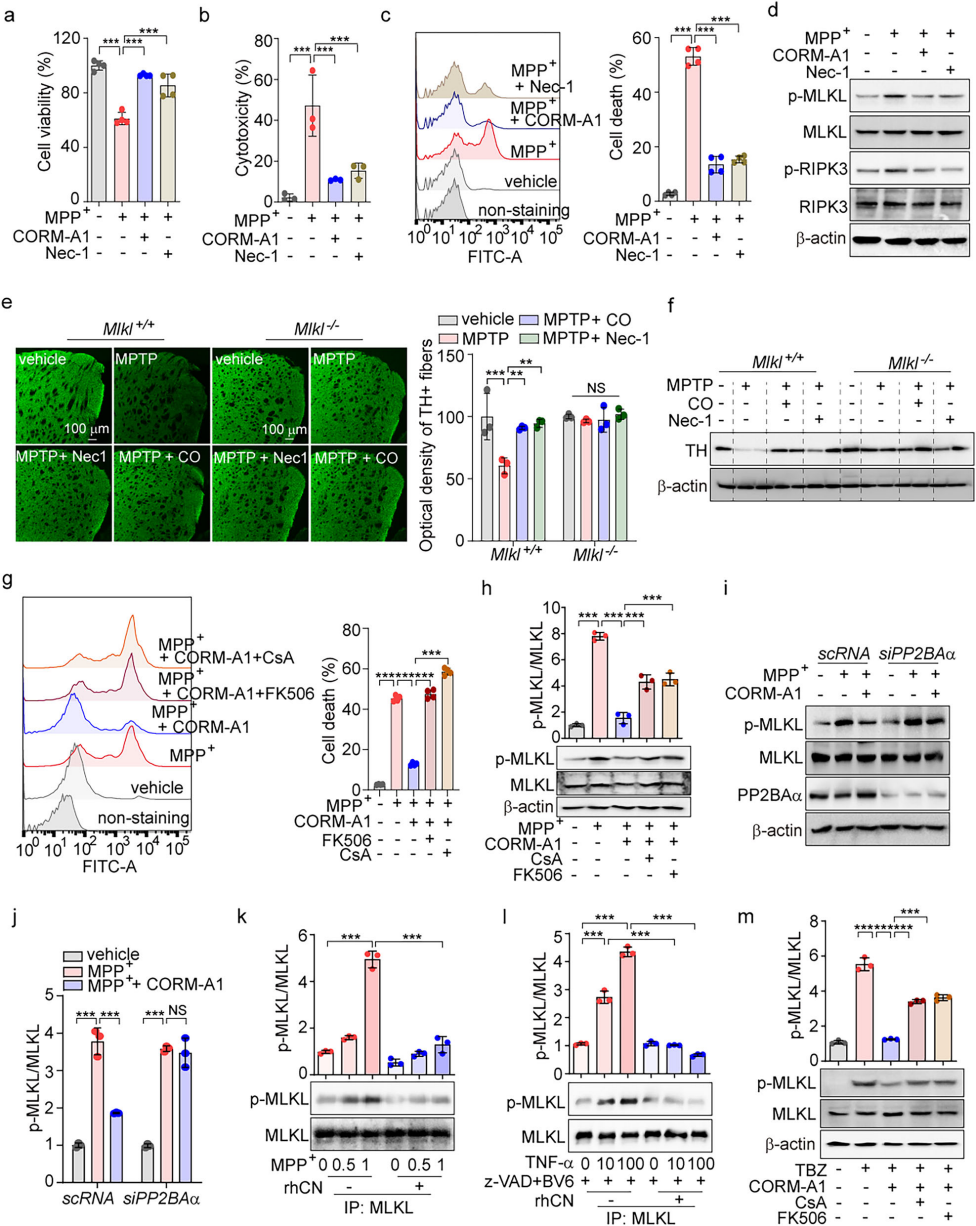
CO-induced calcineurin activity suppresses necroptosis through MLKL Dephosphorylation in a model of neuronal damage caused by MPTP. **a**–**d** SH-SY5Y cells were treated with 20 μM CORM-A1 or 10 μM Nec-1 (necroptosis inhibitor) for 1 h and then treated with 1 mM MPP^+^ for 24 h. **a** Cell viability was determined by WST-8 assay. Necrotic cell death was detected by LDH secretion (**b**) and Sytox green staining (**c**). **d** MLKL phosphorylation was assessed by western blotting. **e**, **f** 8-week-old *Mlkl*^*+/+*^ and *Mlkl*^*−/−*^ mice (*n* = 5) were treated with 250 ppm CO gas by inhalation or with Nec-1 (10 mg/kg, intraperitoneal [i.p.]) for 13 days. Starting on day 4, mice were challenged with MPTP (25 mg/kg, i.p.) for 7 days. Dopaminergic neurons of the striatum region in *Mlkl*^*+/+*^ and *Mlkl*^*−/−*^ mice were determined by tyrosine hydroxylase (TH) staining (**e**). scale bar: 100 μm. The quantification of TH staining is shown in the middle panel. The protein expression levels of TH in the midbrain were detected by western blotting (**f**). **g**, **h** SH-SY5Y cells were pretreated with 20 μM CORM-A1 for 1 h followed by stimulation with 1 mM MPP^+^ for 24 h. After 12 h of treatment, cells were incubated with calcineurin inhibitors, 20 μM Cyclosporin A (CsA) or 10 μM FK506, for 12 h. Necrotic cell death was monitored by Sytox green staining (**g**). Sytox green fluorescence was analyzed by flow cytometry (*left*), and quantification of fluorescence was normalized (*right*). Harvested cells were subjected to western blotting using antibodies against p-MLKL and MLKL (**h**, *left*). Quantification of p-MLKL is shown in the right panel. **i**, **j** SH-SY5Y cells were transfected with scRNA or PP2BAα-targeting siRNA (*siPP2BAα*) and then treated with 20 μM CORM-A1 followed by treatment with 1 mM MPP^+^ for 24 h. MLKL phosphorylation and PP2BAα knockdown were assessed by western blotting (**i**). The quantification of phosphorylated MLKL was analyzed (**j**). **k**, **l** MLKL overexpressing SH-SY5Y cells were treated with 0.5 and 1 mM MPP^+^ (**k**) or 10 and 100 ng/ml TNF-α in the presence of 5 μM BV6 and 20 μM zVAD (**l**) for 24 h or 6 h, respectively. Cell lysates were immunoprecipitated with an anti-MLKL antibody and then incubated with recombinant calcineurin. The reaction mixtures were subjected to western blot analysis with antibodies to p-MLKL and MLKL (*lower panel*). The relative phosphorylated MLKL/MLKL band density ratio was determined (*upper panel*). **m** SH-SY5Y cells were treated with 10 and 100 ng/ml TNF-α in the presence of 5 μM BV6 and 20 μM zVAD (TBZ) for 3 h and then additionally treated with CsA or FK506 for 3 h. The expression of p-MLKL and MLKL was measured by immunoblotting. Quantification of the data is shown in the right panel. Data were expressed as mean ± SD; **p < 0.01, ***p < 0.001, and NS not significant.

Necroptosis, induced by activation of MLKL, promotes neuronal cell death and neuroinflammation in PD [40–42]. CO maintains neuronal differentiation and function by inhibiting cell death [43, 44]. Although CO reduces neuronal damage, its ability to attenuate PD by inhibiting necroptosis remains unclear. To investigate this, we measured cell viability in SH-SY5Y cells following treatment with the dopaminergic neurotoxin 1-methyl-4-phenylpyridinium (MPP^+^) in the presence or absence of either CO-releasing molecule-A1 (CORM-A1), which can release CO in vitro [45], or the necroptosis inhibitor necrostatin-1 (Nec-1). The neurotoxic effects of MPP^+^ reduced cell viability compared to the vehicle-treated group (Fig. 1a). CORM-A1 treatment restored cell viability in SH-SY5Y cells exposed to MPP^+^ (Fig. 1a). In these cells, MPP^+^ treatment induced necrotic cell death, as evidenced by LDH release and Sytox green staining (Fig. 1b, c). Consistent with the cell viability results, CORM-A1 significantly mitigated MPP^+^-induced necrotic cell death (Fig. 1b, c). To investigate whether the inhibitory effects of CO on MPP^+^-induced necrosis are associated with necroptosis, we measured MLKL and RIPK3 phosphorylation. Phosphorylation of MLKL and RIPK3 increased after MPP^+^ treatment, whereas CORM-A1 decreased the phosphorylation of these proteins (Fig. 1d). Similarly, SH-SY5Y cells with Nec-1, a RIPK1 inhibitor, showed comparable results (Fig. 1a–d). To determine whether MLKL expression is responsible for CO-induced neuronal protection in a neurotoxin MPTP-induced mouse model of PD, *Mlkl*^+/+^ and *Mlkl*^−/−^ mice were injected daily with MPTP for seven consecutive days and simultaneously received inhaled CO gas or Nec-1 injections for 13 days. MPTP significantly reduced tyrosine hydroxylase (TH), a dopaminergic neuron marker, in striatal fibers and overall TH expression in *Mlkl*^+/+^ mice, but not in *Mlkl*^-/-^ mice (Fig. 1e). CO reversed the decrease in TH-positive striatal fibers and TH expression caused by MPTP in *Mlkl*^+/+^ mice, but not in *Mlkl*^-/-^ mice (Fig. 1e, f). Similarly, *Mlkl*^+/+^ mice injected with Nec-1, a RIPK1 inhibitor, displayed comparable results.

Induction of cytosolic Ca^2+^ levels by PERK [46] promotes the activation of calcineurin, a Ca2^+^ and calmodulin-dependent serine/threonine phosphatase [47]. We have previously shown that CO-induced PERK activation increases Ca^2+^ levels, potentially leading to calcineurin activation [36]. To determine whether calcineurin is involved in the inhibition of MLKL phosphorylation by CO, we treated SH-SY5Y cells with CORM-A1 and calcineurin inhibitors, cyclosporin A (CsA) or FK506, under MPP^+^-treated conditions. Inhibition of calcineurin by CsA or FK506 abolished the inhibitory effects of CORM-A1 against MPP^+^-induced cell death (Fig. 1g) and MLKL phosphorylation (Fig. 1h). In addition, CORM-A1 reduced MLKL phosphorylation in MPP^+^-treated SH-SY5Y cells transfected with scramble RNA (scRNA). However, this effect was significantly abolished in SH-SY5Y cells transfected with siRNA targeting the calcineurin A catalytic subunit PP2BAα (*siPP2BAα*) (Fig. 1i, j). These data suggest that phosphorylated MLKL may be a direct target of calcineurin. To validate this hypothesis, we immunoprecipitated MLKL from SH-SY5Y cells after MPP^+^ treatment and conducted an in vitro phosphatase assay with recombinant human calcineurin (Fig. 1k). This assay demonstrated that calcineurin can completely dephosphorylate MLKL, confirming MLKL as a substrate for calcineurin. Consistent with these findings, we observed that phosphorylation of MLKL, induced dose-dependently by TNF-α in the presence z-VAD (a pan-caspase inhibitor) and BV6 (a SMAC mimetic) (TBZ), was inhibited by calcineurin (Fig. 1l). Furthermore, CORM-A1 treatment in presence of FK506 or CsA failed to reduce MLKL phosphorylation in response to TBZ [48] (Fig. 1m). We conclude CO protects against neuronal damage by limiting necroptosis through a mechanism involving calcineurin-dependent dephosphorylation of MLKL.

### CO prevents MPTP-induced neurotoxicity via increasing autophagy

**Fig. 2:**
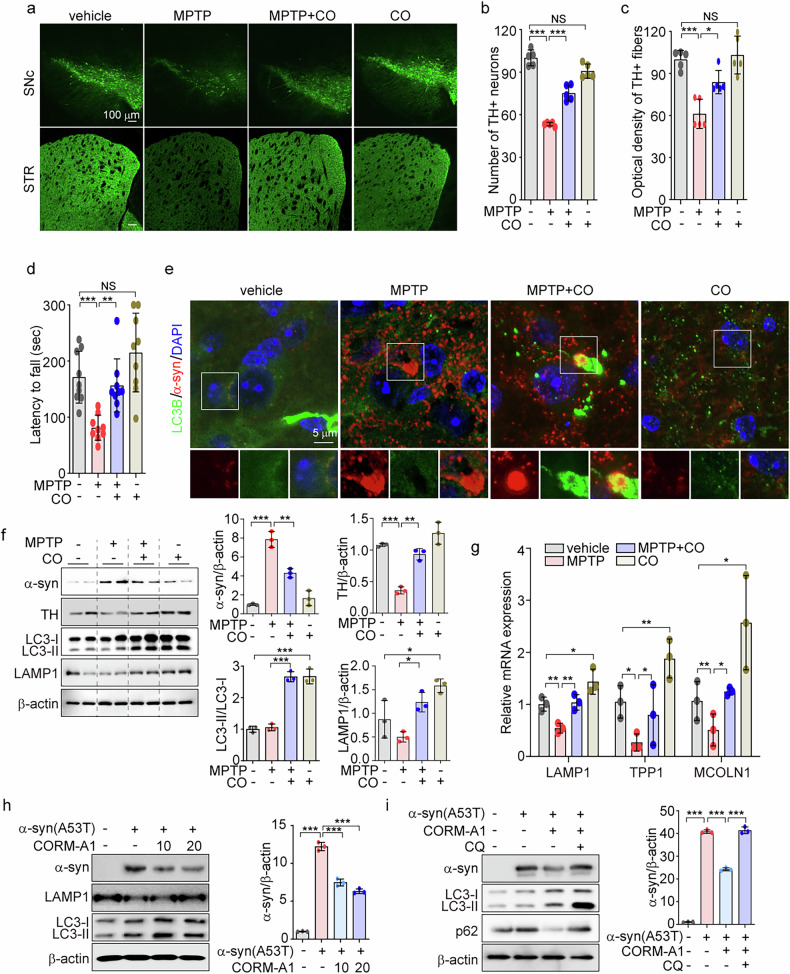
MPTP-induced neurotoxicity is reversed by CO via increasing autophagy. **a**–**g** 8-week-old WT mice (*n* = 5) were pretreated with 250 ppm CO gas (2 h/day) for 13 days. Starting on day 4, mice were subjected to injection of 25 mg/kg MPTP once daily for 7 consecutive days. TH^+^ dopaminergic neurons in SNc and STR were identified by immunofluorescence, scale bar: 100 μm (**a**). The number of TH^+^ dopaminergic neurons in SNc (**b**) and the densitometry of TH immune staining in STR (**c**) were quantified. Locomotor activity of mice was assayed on a rotarod apparatus (**d**). Colocalization of LC3B with α-syn in STR was analyzed by confocal microscopy, scale bar: 5 μm (**e**). **f** The protein expression of α-syn, TH, p-GSK-3β, GSK-3β, LC3B-I/-II, and LAMP1 in the midbrain was detected by western blotting (**f**, *left*), and quantification of the results is shown in the right panel. **g** The mRNA levels of *LAMP1*, *TPP1*, and *MCOLN1* in midbrain were analyzed by qRT-PCR. **h** SH-SY5Y cells were transfected with α-syn-A53T and then treated with 10 and 20 μM CORM-A1 for 12 h. After 12 h, α-syn, LAMP1, and LC3I/II from treated cells were determined by western immunoblotting. **i** SH-SY5Y cells were transfected with α-syn-A53T for 36 h. The cells were treated with 20 μM CORM-A1 for 12 h in the presence or absence of autophagy inhibitor CQ (10 μM). Protein expressions of α-syn, LAMP1, and p62 were detected by western blotting. **h**, **i** Quantification of protein expression is shown in the right panel. Data were expressed as mean ± SD; *p < 0.05, **p < 0.01, ***p < 0.001, and NS not significant.

We have previously shown that CO, when applied at low concentration, exerts protective effects on inflammatory liver injury by stimulating TFEB-regulated mitophagy [36]. However, evidence for amelioration of neurodegeneration by CO remains scarce. We first explored whether CO treatment could attenuate symptoms of neurodegeneration in mice subjected to MPTP. MPTP exposure led to a decrease in TH staining in both the *substantia nigra pars compacta* (SNc) and striatum (STR), with reduced number of TH positive cells and fibers observed in SNc and STR, respectively (Fig. 2a–c). These effects were reversed by CO inhalation (Fig. 2a–c). In addition, MPTP-induced rotarod behavioral deficit in mice was improved by CO inhalation (Fig. 2d).

The aggregation of α-synuclein (α-syn), a pathogenic hallmark of PD, is degraded by the autophagy lysosomal pathway (ALP) [49]. We hypothesized that augmentation of autophagy by CO treatment [36, 50] would attenuate α-syn aggregation in a mouse model of MPTP-induced PD. Confocal microscopy analysis revealed that α-syn levels were higher in the STR of MPTP-treated mice compared to those of vehicle-treated mice. CO inhalation in MPTP-treated mice led to increased colocalization of LC3B with α-syn in the STR (Fig. 2e). Consistent with the immunofluorescence data (Fig. 2a), CO inhalation recovered TH protein levels in the midbrain of MPTP-treated mice. Furthermore, α-syn levels that were increased by MPTP, were lowered by CO inhalation (Fig. 2f). In addition, CO inhalation increased the levels of LC3B-II and LAMP1 in mice subjected to MPTP treatment (Fig. 2f). CO inhalation also restored the mRNA expression levels of lysosome-related genes *LAMP1*, *TPP1*, and *MCOLN1* in the midbrain of MPTP-challenged mice (Fig. 2g). Collectively, our data suggest that autophagy may be required for the protective effects of CO on MPTP-induced neurotoxicity.

To study whether CO can protect against neurotoxicity by inducing autophagy in vitro, we treated the neuroblast-like cell line SH-SY5Y with MPP^+^ or transfected these cells with amyloidogenic gene-expressing plasmids, in the presence or absence of CORM-A1. Treatment with 20 μM CORM-A1 notably reversed MPP^+^-induced cell death in SH-SY5Y cells (Supplementary Fig. 1a). To explore the impact of CO on degradation of α-syn in vitro, we introduced a mutant A53T α-syn (α-syn-A53T) into SH-SY5Y cells and subsequently treated these cells with 10 and 20 μM CORM-A1. The expression levels of α-syn were decreased in response to CORM-A1 treatment (Fig. 2h and i). Consistent with in vivo data (Fig. 2f), CORM-A1 increased LC3B-I/-II conversion and LAMP1 expression in α-syn-A53T cells (Fig. 2h, i). We also evaluated the direct ability of autophagy to degrade the expression of α-syn. LC3B-II and p62 expression in the presence of chloroquine (CQ), an autophagy inhibitor, was greater than that observed in CORM-A1 treated cells in the absence of CQ, while the levels of α-syn and p62 were reduced by CORM-A1 treatment alone (Fig. 2i). The addition of CQ completely inhibited the CORM-A1 mediated degradation of α-syn (Fig. 2i). These findings indicate that CORM-A1 triggers autophagic flux and autophagic degradation of α-syn. Similar to α-syn, amyloid precursor protein (APP) accumulation has been observed in AD [49]. After overexpressing Swedish and Indiana mutant APP (APP^swe/ind^) in SH-SY5Y cells, we found that CORM-A1 prevents aggregation of full-length APP (FL-APP) and increases autophagy markers, LC3B-II and LAMP1 (Supplementary Fig. 1b). Consistent with the data obtained in α-syn-A35T transfected SH-SY5Y cells, we confirmed that CORM-A1 enhances autophagic flux in APP^swe/ind^-transfected cells (Supplementary Fig. 1c). These data indicate that CO ameliorates neurotoxicity in vivo and in vitro, which is mediated by induction of the ALP.

### CO enhances autophagy and inhibits necroptosis through TFEB nuclear translocation in SH-SY5Y cells

**Fig. 3:**
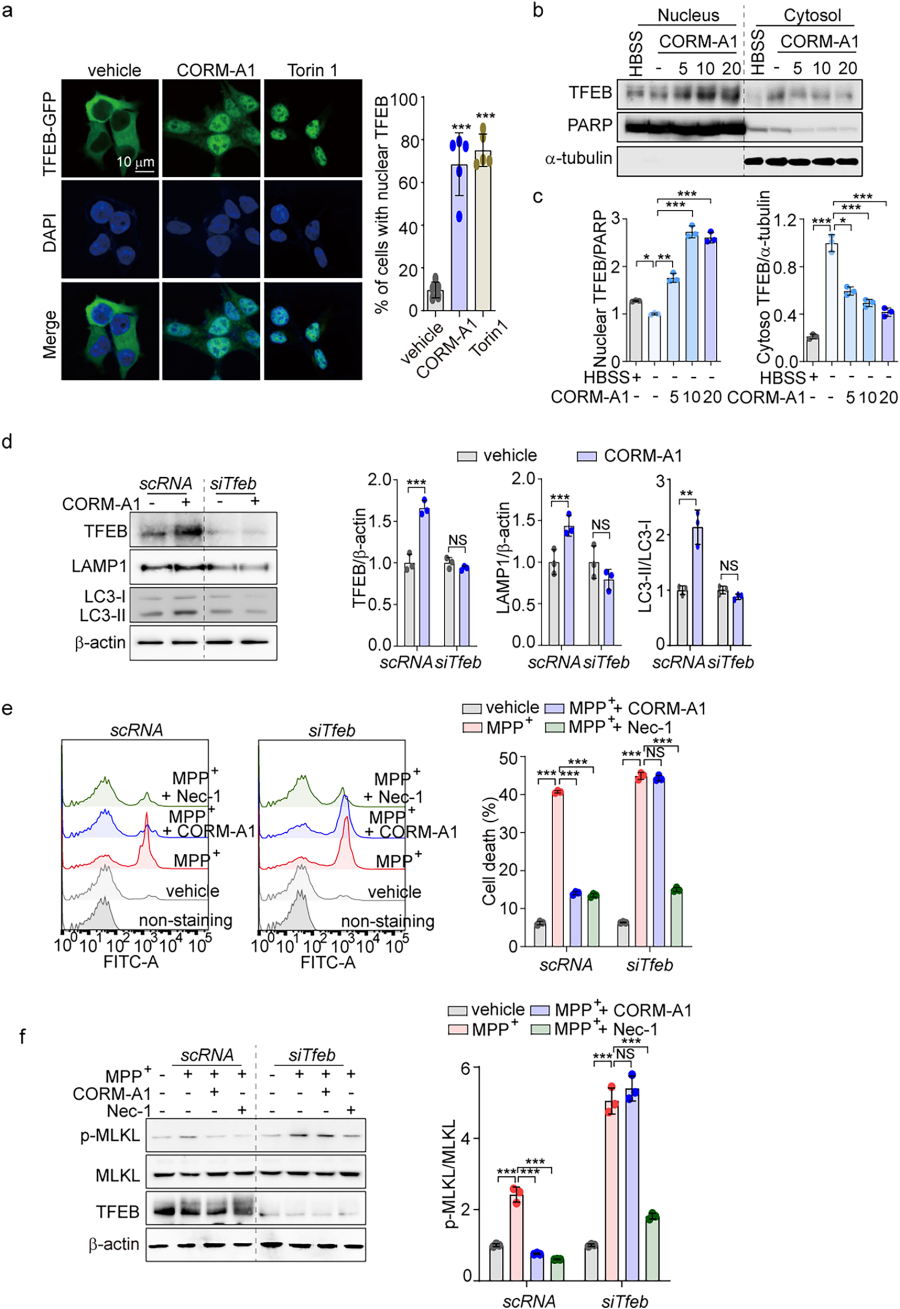
CO enhances autophagy and necroptosis through TFEB nuclear translocation in SH-SY5Y cells. **a** To evaluate the effects of CO on TFEB nuclear translocation, SH-SY5Y cells were transfected with pEGFP-N1-TFEB and then treated with 20 μM CORM-A1 for 6 h and 5 μM Torin1 for 3 h. Cells were detected for fluorescence of GFP using confocal microscopy (*left*), scale bar: 10 μm. The percentage of cells showing nuclear TFEB localization was calculated. *n* > 20 cells per condition (*right*). **b**, **c** SH-SY5Y cells were treated with CORM-A1 at the indicated concentrations (5, 10, and 20 μM) for 6 h and then detected with TFEB antibody in the nuclear and cytoplasmic fractions by western blotting (**b**). The expression of TFEB in nucleus and cytoplasm was quantified (**c**). HBSS was used as a positive control. **d** Levels of TFEB, LAMP1, and LC3-I/-II were detected by western blotting in SH-SY5Y cells transfected with scramble RNA (scRNA) or siRNA against TFEB and then treated with 20 μM CORM-A1 for 12 h. Quantification of band density is shown in the right panel. **e**, **f** SH-SY5Y cells were transfected with scRNA or siRNA against TFEB for 36 h and then treated with 1 mM MPP^+^ in the presence or absence of 20 μM CORM-A1 and 10 μM Nec-1 for 24 h. Necrotic cell death was analyzed by flow cytometry using Sytox green staining (**e**). MLKL phosphorylation and TFEB knockdown were assessed by immunoblotting (**f**). Quantification of fluorescence intensity (**e**) and phosphorylated MLKL expression (**f**) are shown in the right panels. Data were expressed as mean ± SD; *p < 0.05, **p < 0.01, ***p < 0.001, and NS not significant.

We subsequently explored the mechanisms by which CO induces autophagy in SH-SY5Y cells. In our previous study [36], we demonstrated that CO could activate the ALP *via* TFEB in hepatocytes and liver tissues. Using a TFEB-GFP reporter plasmid, we observed that nuclear translocation of TFEB was increased in SH-SY5Y cells by CORM-A1 treatment, and by the mTOR inhibitor, Torin1, used as a positive control for TFEB activation (Fig. 3a). Incubation of these cells with CORM-A1 dose-dependently increased translocation of TFEB from the cytoplasm to the nucleus, as measured by western blotting (Fig. 3b, c). Increased TFEB nuclear translocation in response to CORM-A1 was accompanied by increased activation of the autophagy pathway, measured by LC3B-I/-II conversion, LAMP1 expression (Supplementary Fig. 2a), and increased mRNA levels of the lysosomal genes, *LAMP1* and *MCOLN1* (Supplementary Fig. 2b). To further explore the regulation of autophagic flux by CORM-A1, we analyzed LC3B-II expression in CORM-A1-treated cells in the presence of CQ. Levels of LC3B-II expression were higher for CORM-A1 in combination with CQ than in CORM-A1-treated cells in the absence of CQ, suggesting that CORM-A1 induces autophagic flux (Supplementary Fig. 2c). To confirm the effects of TFEB on CORM-A1-induced autophagy, SH-SY5Y cells were transfected with scramble RNA (*scRNA*) or siRNA targeting TFEB (*siTfeb*) and then incubated with CORM-A1. Knockdown of TFEB expression markedly suppressed CORM-A1-induced expression of TFEB, LAMP1, and LC3B-I/-II conversion (Fig. 3d). Therefore, we propose that CO can promote TFEB nuclear translocation to activate autophagy in SH-SY5Y human neuroblastoma cells.

TFEB is indispensable for autophagy flux as well as suppression of necroptosis [51, 52]. Consequently, we hypothesized that TFEB activation by CORM-A1 could regulate necroptosis. To assess whether necroptosis inhibition by CO related to TFEB, we transfected SH-SY5Y cells with *siTfeb* and then incubated the cells with MPP^+^ in the presence or absence of CORM-A1 and Nec-1. Inhibition of necrotic cell death and phosphorylated MLKL by CORM-A1 was abolished in *siTfeb*-transfected cells (Fig. 3e, f). These data suggest that CO promotes TFEB nuclear translocation to activate autophagy, and that TFEB activation by CO contributes to necroptosis inhibition in SH-SY5Y human neuroblastoma cells.

### CO-induced PERK activation is indispensable for TFEB nuclear import

**Fig. 4:**
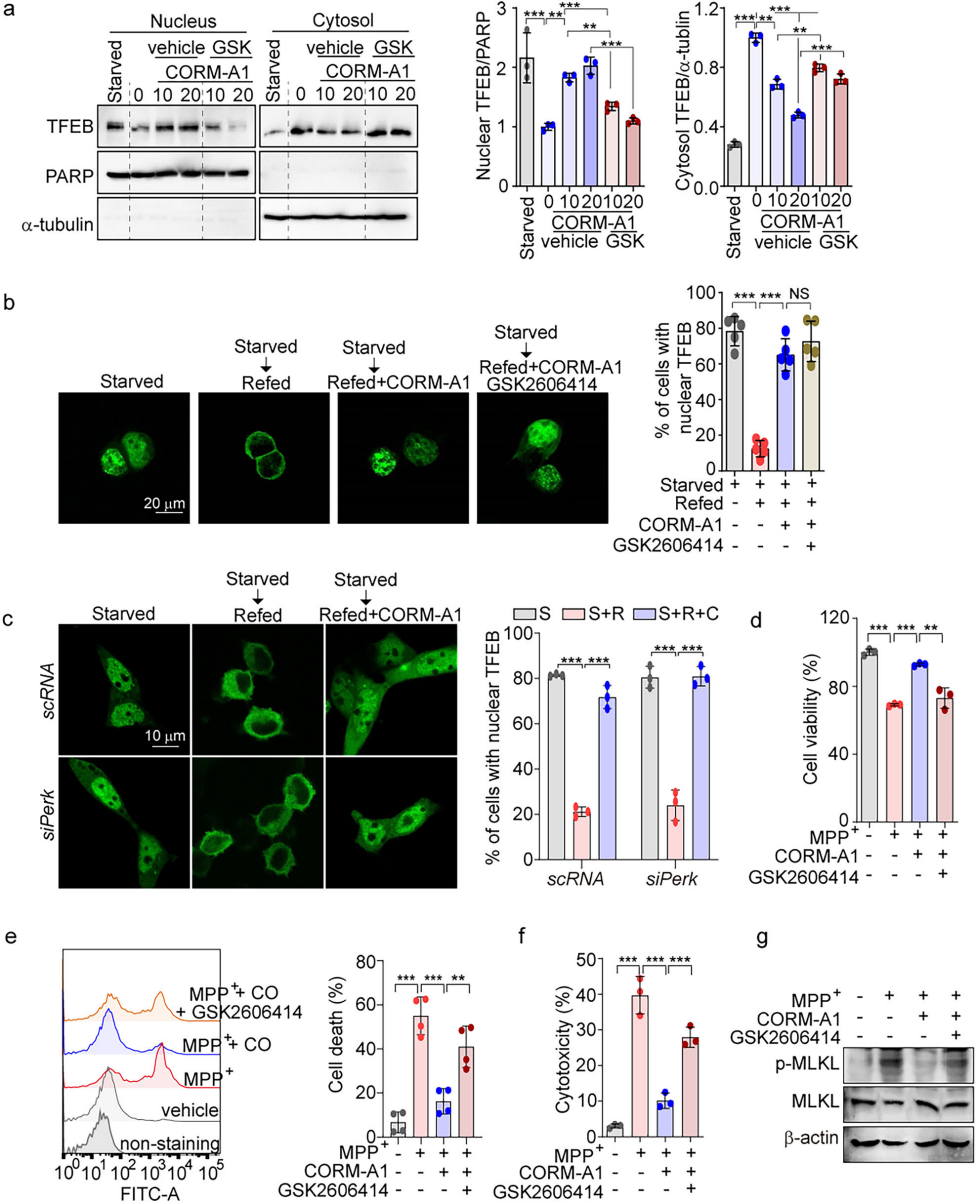
CO-induced PERK activation is indispensable for TFEB nuclear import. **a** SH-SY5Y cells were treated with 10 or 20 μM CORM-A1 for 6 h in the presence or absence of 1 μM GSK2606414 (GSK). SH-SY5Y cells incubated with HBSS medium (Starved) were used as a positive control. Nuclear and cytoplasmic fractions extracted from SH-SY5Y cells were analyzed by western blotting using TFEB antibody (*left*). Quantification of TFEB expression in the nucleus or cytoplasm is shown in right panel. **b**, **c** SH-SY5Y cells were starved using HBSS medium for 2 h and then refed with complete DMEM medium for 2 h in the presence or absence of 20 μM CORM-A1. Images of TFEB-GFP were obtained by confocal microscopy in SH-SY5Y cells treated with 1 μM GSK2606414 PERK inhibitor (**b**) or transfected with siRNA against PERK (**c**), and the percentage of cells showing nuclear TFEB localization was calculated. *n* > 20 cells per condition. S (Starved), S + R (Starved + Refed), S + R + C (Starved + Refed + CORM-A1). **d**–**g** SH-SY5Y cells were treated with 20 μM CORM-A1 for 1 h and then treated with 1 mM MPP^+^ for 24 h in the presence or absence of 1 μM GSK2606414. Cell viability was assessed by WST-8 assay (**d**), and necrotic cell death was detected by Sytox green staining (**e**) and LDH secretion (**f**). MLKL phosphorylation was detected by western blotting (**g**). Data were expressed as mean ± SD; **p < 0.01, ***p < 0.001, and NS not significant.

In previous study, we demonstrated that CO facilitates PERK-dependent lysosomal biogenesis in hepatocytes [36]. Similarly, in SH-SY5Y cells, we found that CORM-A1 enhances PERK phosphorylation in a dose-dependent manner, using thapsigargin (Tg), an inhibitor of the ER Ca^2+^-ATPase, as a positive control (Supplementary Fig. 3a). CORM-A1 treatment also increased p-eIF2α, downstream of PERK, in SH-SY5Y cells, which was reversed by the PERK inhibitor GSK2606414. CORM-A1-induced LC3B-II and LAMP1 levels were also reduced by GSK2606414 (Supplementary Fig. 3b). To explore whether CO-induced PERK activation is involved in ALP activation and TFEB nuclear import, we treated SH-SY5Y cells with GSK2606414, in the presence or absence of CORM-A1. The results show that GSK2606414 inhibits CORM-A1-induced TFEB nuclear translocation (Fig. 4a).

PERK-dependent TFEB nuclear translocation is required for calcineurin activation in hepatocytes [36, 53]. We hypothesized that calcineurin activation, necessary for TFEB dephosphorylation and nuclear import, occurs in CORM-A1 treated SH-SY5Y cells as the result of CO-mediated PERK activation. Treatment with the calcineurin inhibitor FK506 in SH-SY5Y cells reduces both LC3B-I/-II conversion and LAMP1 expression, and reverses TFEB nuclear import induced by CORM-A1 (Supplementary Fig. 3c, d). Similarly, CORM-A1 increased TFEB nuclear translocation in scRNA-transfected cells, whereas this effect was blocked in *siPP2BAα*-transfected cells (Supplementary Fig. 3e). Intriguingly, starvation using HBSS increased nuclear TFEB, which relocated to the cytoplasm upon refeeding. However, CORM-A1 treatment promoted the retention of TFEB within the nuclei of SH-SY5Y cells subjected to media refeeding after starvation (Fig. 4b). Moreover, GSK2606414 did not affect the enhancement of nuclear TFEB induced by CORM-A1 in TFEB-GFP expressing SH-SY5Y cells (Fig. 4b). Similar results were also observed in SH-SY5Y cells transfected with PERK-targeting siRNA (Fig. 4c). These data, taken together, suggest that PERK activation is critical for CO-induced TFEB nuclear import but is not essential for CO-induced nuclear retention. Furthermore, we explored whether CO-PERK activation could mitigate MPP^+^-induced cell death by inhibiting MLKL phosphorylation. CORM-A1 treatment significantly improved the reduction in cell viability caused by MPP^+^ but was ineffective in the presence of GSK2606414 (Fig. 4d). Moreover, MPP^+^-induced necrotic cell death (Figs. 4e, f) and MLKL phosphorylation (Fig. 4g) were decreased by CORM-A1 treatment but not in GSK2606414-treated cells. Based on the data in Fig. 1F, G, it is evident that CO can impede MPP^+^-induced necroptosis through PEPK-mediated calcineurin activation. In summary, CO not only promotes nuclear localization of TFEB but also protects against necroptosis by activating the PERK pathway.

### TFEB nuclear export is inhibited by CO-induced GSK-3β inhibition

TFEB continuously shuttles between the cytoplasm and the nucleus, such that the nuclear import and export of TFEB are crucial for its activation [30]. Inhibition of GSK-3β can decrease the nuclear export of TFEB, resulting in the enhanced retention of nuclear TFEB [31]. We previously found that GSK-3β is phosphorylated at Ser-9 following CO exposure, which causes the inactivation of GSK-3β [38, 54]. Here, we explored whether CO exposure inhibits the nuclear export of TFEB *via* the suppression of GSK-3β in SH-SY5Y cells. First, we demonstrated that CORM-A1 increases GSK-3β phosphorylation, which was not affected by PERK silencing (Supplementary Fig. 4a). Consistent with our previous results in macrophages [38] and hepatocytes [54], CORM-A1 enhanced the phosphorylation of GSK-3β on Ser-9 in a dose-dependent manner in SH-SY5Y cells, while simultaneously reducing the phosphorylation of glycogen synthase (GS), a key target of GSK-3β (Supplementary Fig. 4b). Lithium chloride (LiCl), used as a positive control, also increased p-GSK-3β expression, resulting in a decrease in GS activity (Supplementary Fig. 4b). Further experiments revealed that SH-SY5Y cells, when transfected with constitutively active (CA)-GSK-3β plasmid, showed increased GS phosphorylation. Conversely, transfection with a kinase-dead (KD)-GSK-3β plasmid decreased GS phosphorylation (Supplementary Fig. 4c). However, CORM-A1 reduced p-GS levels in cells transfected with pcDNA3.1 as a control vector, but not in cells transfected with CA-GSK-3β or KD-GSK-3β expression plasmids (Supplementary Fig. 4c). Inhibition of GSK-3β by phosphorylation at Ser9 protects dopaminergic neurons from MPTP challenge [55]. While inhibitory phosphorylation of GSK-3β was downregulated by MPTP challenge, CO inhalation increased GSK-3β phosphorylation in midbrain (Supplementary Fig. 4d). In addition, CORM-A1 rescued cell viability in MPP^+^-challenged SH-SY5Y cells. However, CORM-A1 did not rescue cell viability in CA-GSK-3β-transfected cells (Supplementary Fig. 4e).

**Fig. 5:**
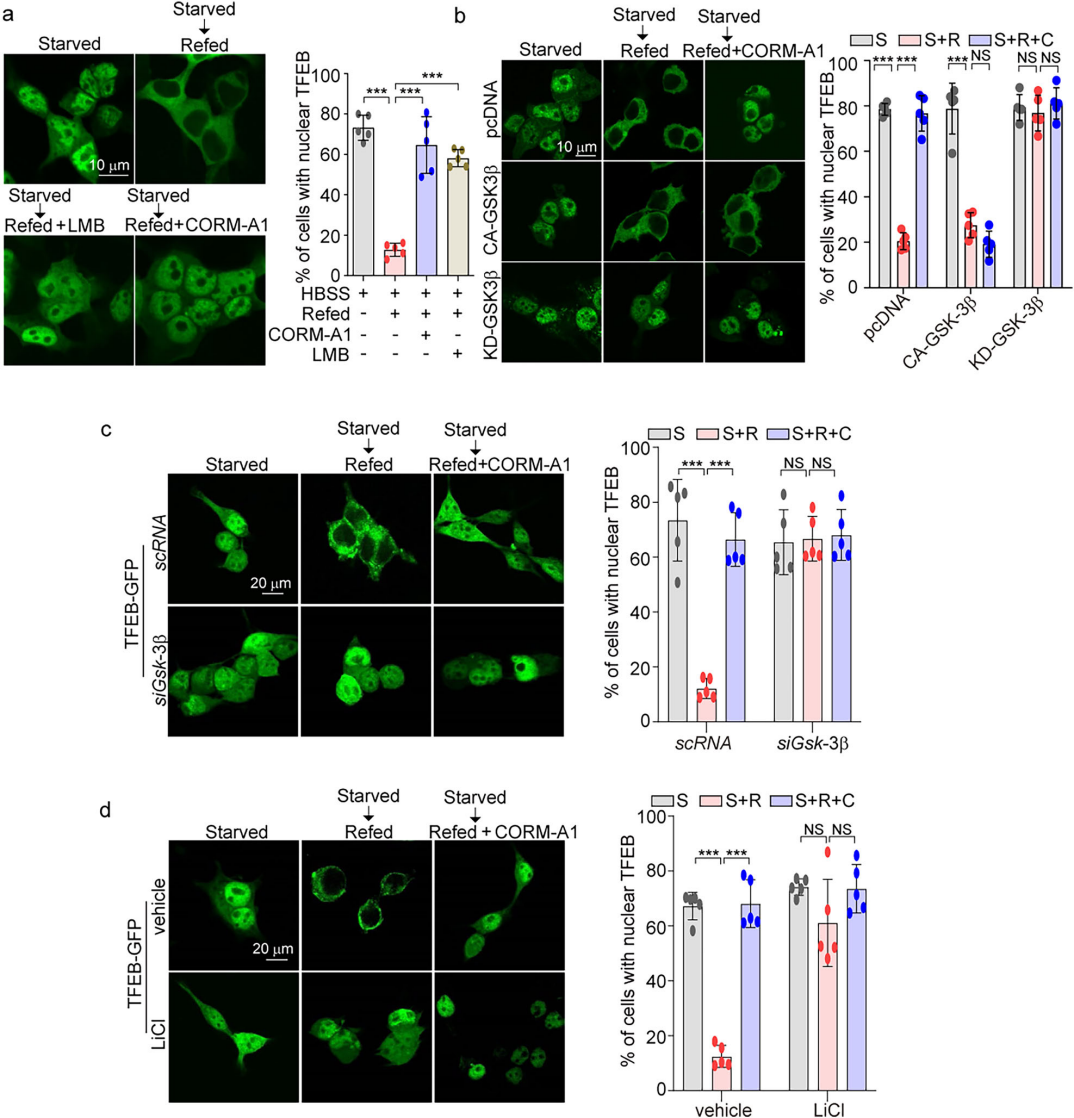
TFEB nuclear export is prevented by CO-induced GSK-3β inhibition. **a** To assess the effect of CO on inhibition of TFEB nuclear export, SH-SY5Y cells transiently expressing TFEB-GFP were starved for amino acids for 2 h and then refed with complete DMEM for 2 h in the presence or absence of 20 μM CORM-A1 and the nuclear export inhibitor LMB (20 nM) and analyzed by confocal microscopy. **b** SH-SY5Y cells were co-transfected with TFEB-GFP, CA-GSK-3β, or KD-GSK-3β. The transfected cells were treated with 20 μM CORM-A1 for 2 h after cells were refed with complete DMEM. Fluorescence images of SH-SY5Y cells were obtained by confocal microscopy. S (Starved), S + R (Starved + Refed), S + R + C (Starved + Refed + CORM-A1). **c**, **d** SH-SY5Y cells were placed in HBSS medium for 2 h and then refed with complete DMEM medium for 2 h in the presence or absence of 20 μM CORM-A1. Images of TFEB-GFP were detected by confocal microscopy in SH-SY5Y cells transfected with siRNA against GSK-3β (**c**) or treated with LiCl (**d**). The percentage of cells showing nuclear TFEB localization was calculated. *n* > 20 cells per condition. Data were expressed as mean ± SD; ***p < 0.001 and NS not significant.

To investigate the effect of CO on TFEB nuclear export, SH-SY5Y cells were starved in an amino acid-free media containing glucose (HBSS) and then were refed with complete medium. In SH-SY5Y cells transfected with TFEB-GFP, TFEB entered the nucleus in response to incubation in HBSS media and returned to the cytoplasm on refeeding with complete medium (Fig. 5a). However, TFEB nuclear export triggered by refeeding with complete medium after starvation was significantly inhibited by CORM-A1 or leptomycin B (LMB), an inhibitor of nuclear export (Fig. 5a). To examine whether the inhibition of GSK-3β by CORM-A1 was required for nuclear retention of TFEB, we transfected SH-SY5Y cells with expression plasmid for CA-GSK-3β or KD-GSK-3β⊡ Unlike cells transfected with control vector, overexpression of CA-GSK-3β prevented the CORM-A1-dependent retention of nuclear TFEB after refeeding (Fig. 5b). Refeeding with complete media did not promote TFEB nuclear export in SH-SY5Y cells expressing KD-GSK-3β in either the presence or absence of CORM-A1 (Fig. 5b). Consistent with the effects of KD-GSK-3β transfection, in cells transfected with GSK-3β-targeting siRNA (Fig. 5c) or treated with LiCl, a GSK-3β inhibitor (Fig. 5d), refeeding with complete media had no effect on TFEB nuclear export in either the presence or absence of CORM-A1. These results indicate that CO-dependent inhibition of GSK-3β is sufficient to prevent TFEB nuclear export, leading to sustained TFEB levels in the nucleus.

### Regulation of GSK-3β and PERK by CO synergistically sustains nuclear TFEB levels, leading to suppressed amyloidogenesis

**Fig. 6:**
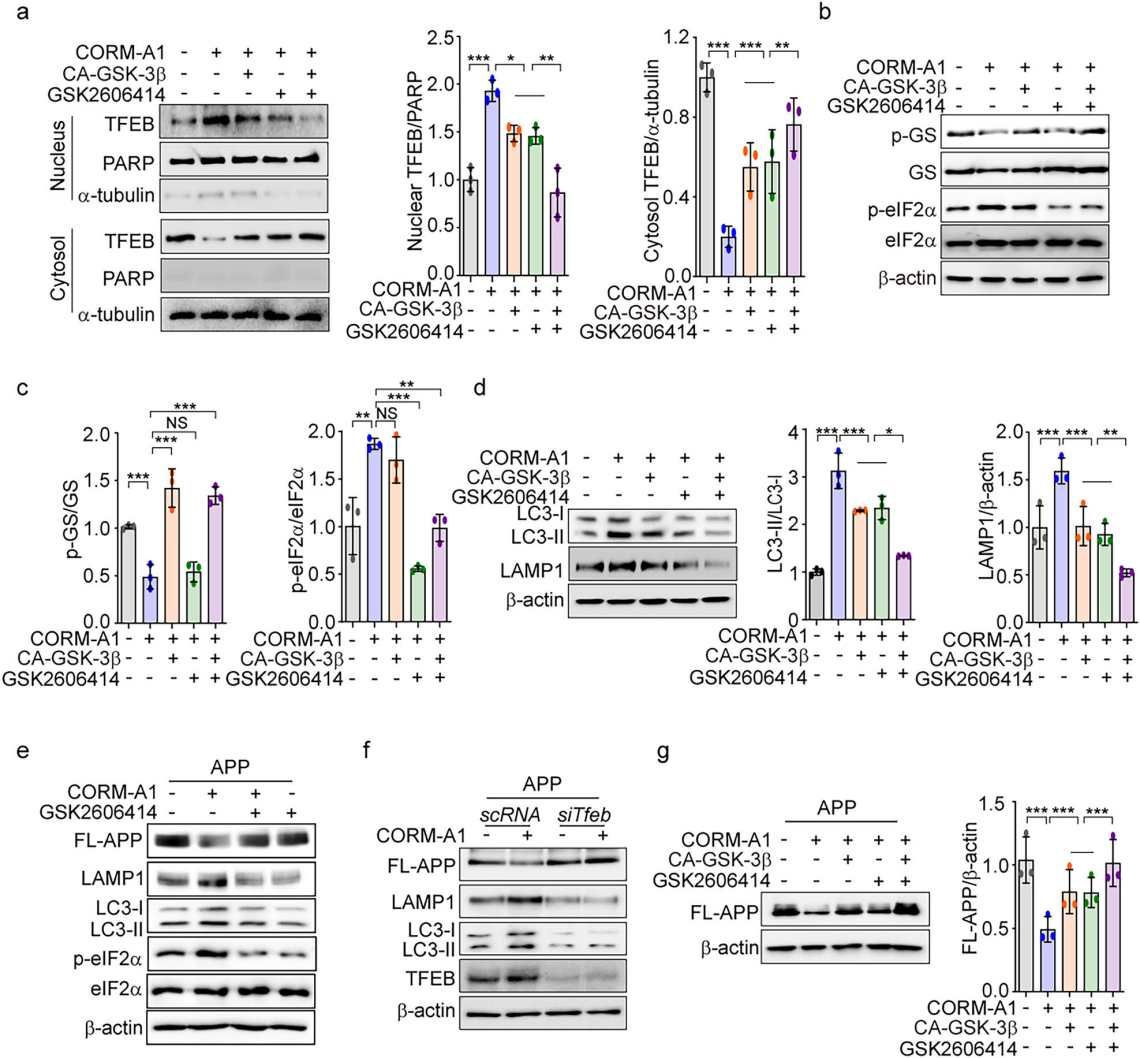
Regulation of GSK-3β and PERK by CO sustain nuclear TFEB levels, leading to suppressed amyloidogenesis. **a**–**d** SH-SY5Y cells were treated with 20 μM CORM-A1 after the cells were transfected with CA-GSK-3β or treated with 1 μM GSK2606414. Nuclear and cytoplasmic fractions extracted from SH-SY5Y cells were analyzed by western blotting using TFEB antibody (l*eft*), and quantification of nuclear TFEB and cytosol TFEB was analyzed (*right*) (**a**). The levels of p-GS, GS, p-eIF2α, and eIF2α expression were assessed by western blotting (**b**). Quantification of p-GS and p-eIF2α (**c**). The protein expression of autophagy protein LC3-I/-II and lysosomal protein LAMP1 was detected by western blotting (*left panel*) (**d**), and quantification of LC3-II conversion and LAMP1 is shown in the *right panels*. **e** SH-SY5Y cells were transfected with APP-Swe/Ind and then treated with 20 μM CORM-A1 for 12 h in the presence or absence of 1 μM GSK2606414. The levels of APP, LAMP1, LC3-I/-II, p-eIF2α, and eIF2α expression were detected by western blotting. **f** SH-SY5Y cells were co-transfected with APP-Swe/Ind and si*Tfeb* for 36 h and were treated with 20 μM CORM-A1 for 12 h. The levels of APP, LAMP1, LC3-I/-II, and TFEB were analyzed by western blotting. **g** SH-SY5Y cells were treated with 20 μM CORM-A1 after the cells were co-transfected with CA-GSK-3β and APP-Swe/Ind or treated with 1 μM GSK2606414. The protein expression of FL-APP was determined by western blotting (*left*), and quantification of FL-APP was analyzed (*right*). Data were expressed as mean ± SD; *p < 0.05, **p < 0.01, ***p < 0.001, and NS not significant.

To explore whether the sustained nuclear presence of TFEB is influenced by the synergic effects of GSK-3β and PERK, we treated CA-GSK-3β-transfected SH-SY5Y cells with GSK2606414 in the presence of CORM-A1. The combination of GSK-3β activation (using CA-GSK-3β) and PERK inhibition (with GSK2606414) significantly reduced nuclear TFEB accumulation more efficiently than either GSK-3β activation or PERK inhibition alone (Fig. 6a). Additionally, CORM-A1-induced reduction in pGS levels or increases in p-eIF2α were blocked in cells expressing CA-GSK-3β or treated with GSK2606414, respectively (Fig. 6b, c). Moreover, the CO-mediated increases in LC3B-II and LAMP1 expression were abolished by GSK-3β activation or PERK inhibition, and these effects were more pronounced when both were combined (Fig. 6d).

We further investigated whether the synergistic effects of GSK-3β and PERK regulation following CO treatment could ameliorate amyloidogenesis in SH-SY5Y cells transfected with APP-Swe/Ind. We found that GSK2606414 treatment impaired the ability of CO to reduce full-length APP (FL-APP) expression and to enhance LC3B-II and LAMP1 expression (Fig. 6e). In addition, APP expression was higher in cells transfected with si*Tfeb* compared to scRNA-transfected cells (Fig. 6f). Moreover, the reduction of APP expression by CORM-A1 was abrogated by TFEB knockdown (Fig. 6f). Silencing of TFEB also decreased the expression of LC3B-II and LAMP1 compared to scRNA-transfected cells, in both the presence and absence of CORM-A1 (Fig. 6f). These results suggest that CO-induced degradation of amyloidogenic protein is mediated through the maintenance of nuclear TFEB levels by the cooperative activation of PERK and inhibition of GSK-3β (Fig. 6g). Also, the CORM-A1-mediated rescue of cell viability in MPP^+^-treated cells was diminished by either GSK-3β activation or PERK inhibition (using GSK2606414), and further diminished by combination of both interventions (Supplementary Fig. 4f). We conclude that these combined effects of CO might offer potential therapeutic benefits in reducing amyloidogenesis and neurotoxin-induced damage associated with neurogenic diseases.

### CO-induced Beclin 1 expression protects against neuronal cell death by preventing necroptosis via suppressing MLKL oligomerization

**Fig. 7:**
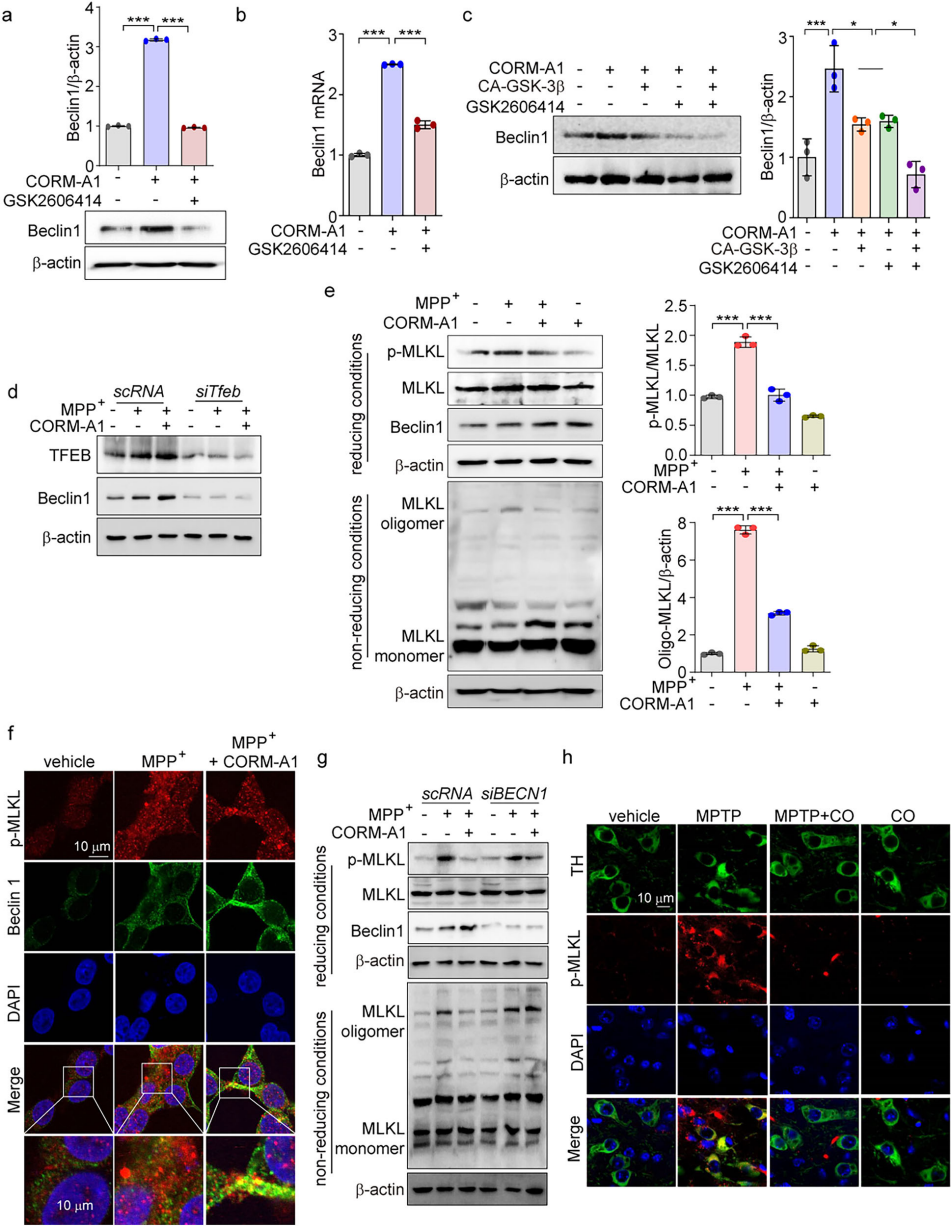
CO-induced Beclin 1 protects against neuronal cell death by preventing necroptosis via suppressing MLKL oligomerization. **a**, **b** SH-SY5Y cells were pretreated with 1 μM GSK2606414 for 30 min and then treated with 20 μM CORM-A1 for 12 h. Beclin 1 protein expression (**a**) and Beclin 1 mRNA expression (**b**) were determined by western blotting and qRT-PCR, respectively. **c** Beclin 1 expression was detected by western blotting in SH-SY5Y cells treated with 20 μM CORM-A1 after the cells were transfected with CA-GSK-3β or pretreated with 1 μM GSK2606414 (*left*). Quantification of Beclin 1 is shown in the *right panel*. **d** SH-SY5Y cells were transfected with scRNA or si*Tfeb* and then treated with 1 mM MPP^+^ and 20 μM CORM-A1 for 24 h. The levels of TFEB and Beclin 1 protein expression were analyzed by western blotting. **e** SH-SY5Y cells were treated with 1 mM MPP^+^ for 24 h after pretreatment with 20 μM CORM-A1 for 1 h. Reducing samples were subjected to western blotting for detection of p-MLKL, MLKL, and Beclin 1. To confirm MLKL oligomerization, analysis of non-reducing samples was performed by western blotting using an MLKL antibody. **f** SH-SY5Y cells were pretreated with 20 μM CORM-A1 for 2 h, followed by treatment with 1 mM MPP^+^ for 24 h. Cells were immunostained with anti-p-MLKL and anti-Beclin 1 antibodies, and counterstained with DAPI. **g** SH-SY5Y cells were transfected with scRNA or *siBECN1* and then treated with 1 mM MPP^+^ for 24 h in the presence or absence of 20 μM CORM-A1. Reducing samples were subjected to western blotting for detection of p-MLKL, MLKL, and Beclin 1. To confirm MLKL oligomerization, analysis of non-reducing samples was performed by western blotting using an MLKL antibody. **h** Mice (*n* = 5) were subjected to MPTP (25 mg/kg) injection in the presence or absence of CO inhalation (250 ppm) for 13 days. Starting on day 4, mice were treated with MPTP once daily for 7 consecutive days. TH and p-MLKL expression in SNc were determined by immunofluorescence, scale bar: 10 μm. Data were expressed as mean ± SD; *p < 0.05 and ***p < 0.001.

Recent studies indicate that necroptosis, a regulated form of cell death, can contribute to the pathogenesis of neurodegenerative diseases [16, 56]. Moreover, Beclin 1, an autophagy protein, can regulate the oligomerization of MLKL, a crucial component in necroptosis [20]. To demonstrate that CO can induce Beclin 1, we measured its expression levels in SH-SY5Y cells treated with CORM-A1. CORM-A1 treatment elevated the expression of Beclin 1 protein in a dose-dependent manner, a response also observed with Torin-1, an mTOR inhibitor, used as a positive control (Supplementary Fig. 5a). Knockdown of TFEB reversed the increase in Beclin 1 protein and mRNA in response to CORM-A1 treatment in SH-SY5Y cells (Supplementary Fig. 5b, c). Chemical inhibition of PERK using GSK2606414 also prevented the induction of Beclin 1 protein and mRNA by CORM-A1 treatment (Fig. 7a, b). An increase of Beclin 1 by CORM-A1 was synergistically abolished by GSK-3β activation and PERK inhibition (Fig. 7c), consistent with effects on other ALP genes such as LC3B-II or LAMP1 (Fig. 6d). Thus, we suggest that CO enhances Beclin 1 expression through the cooperative effect of GSK-3β activation and PERK inhibition.

Beclin 1 has been demonstrated to inhibit MLKL oligomerization without affecting MLKL phosphorylation, thereby inhibiting necroptosis [20]. We thus investigated whether CO-induced Beclin 1 can inhibit necroptosis through the reduction of MLKL oligomerization in MPP^+^-treated SH-SY5Y cells. To first confirm the role of Beclin 1 in CO-mediated inhibition of MPP^+^-induced cell death, we transfected SH-SY5Y cells with sc*RNA* or si*Tfeb* and challenged them with MPP^+^. CORM-A1 treatment induced Beclin 1 expression in sc*RNA*-transfected cells but not in si*Tfeb*-transfected cells challenged with MPP^+^ (Fig. 7d). We also found that CO suppressed both the phosphorylation and oligomerization of MLKL, and increased Beclin 1 expression in MPP^+^-treated cells (Fig. 7e). Consistent with previous report [20], CORM A1-induced Beclin 1 protein colocalized with p-MLKL (Fig. 7f). MLKL phosphorylation increased in MPP^+^-treated SH-SY5Y cells transfected with scRNA. In *siBECN1*-transfected SH-SY5Y cells treated with MPP^+^, CORM-A1 slightly decreased the level of phosphorylated MLKL, whereas inhibition of MLKL oligomerization by CORM-A1 was markedly abolished (Fig. 7g). In vivo, mice treated with MPTP exhibited higher levels of p-MLKL in the SNc compared to vehicle-treated mice, whereas CO inhalation reduced MPTP-induced p-MLKL expression (Fig. 7h). These results suggest that CO decreased necroptosis in neuronal cells and in the SNc of mice induced by MPTP or MPP^+^, by a mechanism that involved Beclin 1 regulation of p-MLKL.

## Discussion

This study has uncovered novel mechanisms underlying the neuroprotective effects of CO in models of neuronal cell injury and neurodegenerative diseases, particularly through its modulation of necroptosis and autophagy pathways. There are several principal findings: (i) the CO-PERK-Calcineurin pathway suppresses necroptosis by promoting the dephosphorylation of MLKL (ii) CO enhances autophagy for neuroprotection by synergistically increasing nuclear TFEB through modulation of PERK/GSK-3β. (iii) Sustained nuclear TFEB accumulation by CO increases Beclin 1 expression, which prevents necroptosis by blocking MLKL oligomerization.

Necroptosis is associated with neuronal cell death in various neurodegenerative diseases, such as PD and AD [16, 56]. Necroptosis is induced in a pathway involving RIPK1/RIPK3, and MLKL, in which MLKL is phosphorylated by RIPK3, a downstream component of TNF-induced necroptosis; and phosphorylated MLKL in turn undergoes oligomerization, which mediates plasma membrane permeabilization [57]. Therefore, MLKL dephosphorylation is crucial for inhibition of necroptosis.

We found that the CO-PERK-calcineurin pathway dephosphorylates MLKL to prevent necroptosis in a mouse model of PD. Moreover, the inhibition of MLKL oligomerization led to inhibition of necroptosis. A recent study demonstrated that Beclin 1, an autophagy protein, suppresses necroptosis through interacting with MLKL, which results in reduced MLKL oligomerization, in an autophagy-independent manner and that Beclin 1 selectively inhibits MLKL oligomerization, but not MLKL phosphorylation [20]. Our previous findings demonstrated that CO at low concentration promotes TNF-α degradation by tristetraprolin activation [37, 49]. Based on these data, we suggest that CO may also inhibit MLKL phosphorylation by suppression of TNF-α production to confer inhibitory effects on MPP^+^-induced necroptotic cell death in SH-SY5Y cells.

In our studies we demonstrate that either CORM-A1 or the necroptosis inhibitor Nec-1, can protect against neuronal cell death. Both treatments resulted in the inhibition of MLKL phosphorylation, which is a hallmark of necroptosis. Nec-1 directly inhibits the upstream regulator RIPK1, which, in addition to inhibition of necroptosis, can potentially regulate multiple pathways, including apoptosis. Thus, we cannot exclude the possibility that modulation of necroptosis-independent pathways by RIPK1, such as apoptosis, could contribute to neuronal injury and cell death in our model. Further research will be required to determine the precise role of RIPK1 in neuronal cell death in this model, and its role as a target for the cytoprotective effects of CO.

Among the three mammalian UPR pathways: PERK, ATF6, and IRE1, we have previously shown that CO selectively activates the PERK branch of the UPR through enhanced production of mitochondrial reactive oxygen species, but does not affect IRE1 and ATF6 [58, 59]. Moreover, from our previous results, CO-activated PERK induced the nuclear translocation of TFEB in hepatocytes [36]. Recent studies suggest that TFEB undergoes nuclear-cytoplasmic shuttling [30, 31]. GSK-3β inhibition, mediated by phosphorylation at Ser-9, activates autophagy and TFEB [55]. GSK-3β phosphorylates Ser-138 on the TFEB nuclear export signal (NES), which mediates TFEB nuclear export [31].

Our current data demonstrates that CO-dependent PERK activation induces TFEB nuclear import in SH-SY5Y cells whereas PERK inhibition using GSK2606414 prevents TFEB translocation to the nucleus. In contrast, our results demonstrate that PERK is not required for inhibition of TFEB nuclear export. Further, once TFEB is translocated, its retention in the nucleus is independent of PERK and is instead regulated by other factors such as GSK-3β which regulates nuclear export. We have shown that combination of GSK-3β activation and PERK inhibition completely reversed the cytoprotective effects of CORM-A1 in this model, and also effectively reduced TFEB nuclear accumulation and LC3B-II accumulation. We also show that GSK-3β was inhibited by CO in the midbrain tissues of mice and in SH-SY5Y cells and that this effectively suppressed TFEB nuclear export. Taken together, these results suggest that GSK-3β inhibition and PERK activation by CO produce synergic effects on retention of nuclear TFEB through activation of TFEB nuclear import and inhibition of TFEB nuclear export. We also conclude that, while PERK inhibition prevents CO-induced TFEB translocation, TFEB nuclear retention and export is primarily regulated by GSK-3β.

Our current studies strongly implicate regulation of TFEB as a contributor to cytoprotection afforded by CO. In contrast to protection afforded by Nec-1, which was not affected by TFEB knockdown, the neuroprotective effects of CO were dependent on TFEB, a master regulator of autophagy. Further, we have shown that the autophagy protein Beclin 1, which is regulated by TFEB, can cross talk to the regulation of necroptosis, primarily through the downregulation of MLKL oligomerization. Although we have shown colocalization of Beclin 1 and MLKL in neural cells, our results do not confirm a direct binding interaction. Since autophagy is subject to complex regulatory pathways involving MTOR and AMPK signaling and others, we cannot exclude that activation of autophagy in our model by CO involved additional mechanistic pathways beyond TFEB regulation.

Autophagy and necroptosis are cellular processes that have attracted particular interest as mechanistic targets in select neurodegenerative diseases. To prevent neurodegeneration and neurological disabilities in neurodegenerative disorders, it will be important to study the mechanisms that control amyloidogenesis as well as neuronal cell loss. In MPTP-induced PD models, inhibition of MLKL protein-mediated necroptosis can significantly ameliorate disease pathogenesis [40]. Our data show that CO ameliorates the motor phenotype, rescues dopaminergic neurons, and degrades α-syn, a key amyloidogenic protein, in the MPTP-induced PD mouse model. Moreover, we show that CO can prevent MPP^+^-induced cell death. Furthermore, α-syn and FL-APP, central proteins in AD, can be degraded in response to CO treatment, as shown in mutant α-syn and APP expressing SH-SY5Y cells, which suggest activation of the ALP. Therefore, we conclude that the CO-PERK-calcineurin pathway ameliorates neurodegenerative diseases via regulating autophagy and necroptosis.

In summary, our data demonstrate that the increased activity of PERK-calcineurin and the inhibition of GSK by CO effectively regulate the nuclear-cytosolic shuttling of TFEB, not only increasing autophagy but also inhibiting necroptosis via induction of Beclin 1, and that the PERK-calcineurin pathway also inhibits necroptosis through dephosphorylation of MLKL. Thus, low dose application of CO may represent a promising therapeutic strategy for neurodegenerative diseases.

## Materials and methods

### Reagents

The CO releasing molecule, sodium boranocarbonate (CORM-A1), the PERK inhibitor (GSK2606414), the calcineurin inhibitors Cyclosporin A (CsA) and FK506, MPP^+^ (1-N-methyl-4-phenylpyridinium), leptomycin B (LMB), lithium chloride (LiCl), necroptosis inhibitor Necrostatin-1 (Nec-1), SMAC mimetic BV-6, and MPTP (1-methyl-4-phenyl-1, 2, 4, 3, 6-tetrahydropyridine) were purchased from Sigma-Aldrich (St. Louis, MO, USA). Torin1 was from Tocris Bioscience (Minneapolis, MN, USA). Recombinant human TNF-α was from Peprotec (Hartford, CT, USA). The apoptosis inhibitor zVAD-FMK was acquired form Bachem (Heidelberg, Germany). As a negative control, CORM-A1 was dissolved in 0.1 M HCl and bubbled with pure N_2_ [45].

### Animals

All experiments with mice were approved by the Animal Care Committee of the University of Ulsan (HTC-19-010). Wild type C57BL/6 mice (6 weeks old, male) were acquired from ORIENT (Busan, Korea), and *Mlkl* knockout (KO) mice were purchased from Cyagen Bioscience (Suzhou, China). These mice were allocated to experimental groups in a general manner without the use of a specific randomization method and were randomly divided into five groups (*n* = 5 in each group, vehicle, MPTP, MPTP + CO, MPTP+Nec-1, and CO-treated). Grouping was performed to ensure comparable baseline characteristics among groups. Blinding was not applied in this study. Mice (8 weeks old, male) were subjected to inhalation of CO at 250 parts per million (ppm) in air (Core Gas, Ulsan, Korea) for 2 h per day in a sealed exposure chamber (LB Science, Daejeon, Korea) at room temperature or intraperitoneal (*i.p*.) administered with 10 mg/kg Nec-1 for 13 days. Starting on day 4, mice received *i.p*. injections of vehicle or MPTP at a dose of 25 mg/kg once daily for 7 consecutive days, as described previously [60]. After 13 days, mice were euthanized using Avertin. Whole brain was perfusion-fixed in 10% neutral-buffered formalin solution (Sigma-Aldrich) as outlined in prior studies [61]. Subsequently, the brain tissues were submerged in 30% sucrose and preserved at 4 °C until processing for immunofluorescence. Tissues from mouse midbrain were extracted via western blotting and RT-PCR.

### Rotarod test

To test motor ability, each mouse was placed on a rod with 30 mm diameter rotating at a constant speed of 10 rpm for a maximum of 3 min. Mice were subjected to four trials 1 day before the test to familiarize themselves with the task. On the following day, mice were given four trials with increasing rotation from 4 rpm to 40 rpm, as previously described [62], recording their latency to fall. The cutoff time was set at 5 min. The results from three experiments were used for statistical analysis.

### Cell culture

The human neuroblastoma cell line SH-SY5Y (ATCC, CRL-2266) was maintained in DMEM (Gibco, Grand Island, USA) supplemented with 10% FBS (Gibco, Melbourne, Australia) and 1% penicillin-streptomycin (Gibco, Grand Island, USA). Cells were incubated at 37 °C in humidified incubators containing an atmosphere of 5% CO_2_.

### WST-8 assay

To measure cell viability, SH-SY5Y cells were seeded in 96-well plates at a density of 5000 cells/well, with three replicate wells per group. After reaching 70% confluence, the cells were exposed to 1 mM MPP^+^ for 24 h after pretreatment with 20 μM CORM-A1 for 2 h. The cells were incubated with WST-8 (Biomax, Seoul, Korea) for 1 h. To quantify cell viability, the optical density of samples was read at 450 nm on a SpectraMax iD3 (Molecular Devices, Sunnyvale, CA, USA).

### Transfection

To knockdown mRNA levels of *TFEB*, *GSK-3β*, *PERK*, *BECN-1*, and *PP2BAα*, SH-SY5Y cells were transfected with siRNA against *TFEB*, *GSK-3β*, *PERK*, *BECN-1*, and *PP2BAα* (Santa Cruz, CA, USA), or with scramble siRNA (scRNA) (Ambion, Austin, TX, USA) as the control, using Lipofectamine 2000 (Invitrogen, Carlsbad, CA, USA), according to the manufacturer’s protocol. α-syn-A53T, pCAX-APP-Swe/Ind, pEGFP-N1-TFEB, pcDNA3.1, pcDNA3.1-GSK-3β-S9A, and pcDNA3.1-GSK-3β-K85A, and hMLKL-Venus were from Addgene (Watertown, MA, USA) and were transfected into cells *via* Lipofectamine 2000 (Invitrogen).

### qRT-PCR

Total RNA from cells and midbrain tissues was isolated by QIAzol Lysis reagent (QIAGEN, Valencia, CA, USA). Total RNA was used to synthesize cDNA by using M-MLV reverse transcriptase (Promega, Madison, WI, USA). The cDNA product was subjected to real-time quantitative PCR (qRT-PCR). qRT-PCR was performed with SYBR Green Master Mix on an ABI 7500 Fast Real-Time PCR System (Applied Biosystems, Foster City, CA, USA). The following qRT-PCR primers were used: mouse GAPDH (f-cggcctcaccccatttg, r-gggaagcccatcaccatct), mouse MCOLN1 (f-gcgcctatgacaccatcaa, r-tatcctggactgctcgat), mouse TPP1 (f-aagccaggcctacatactcaga, r-ccaagtgcttcctgcagtttaga), mouse LAMP1 (f-taatggccagcttctctgcctcctt, r-aggctggggtcagaaacattttctt), human GAPDH (f-caatgaccccttcatcctc, r-agcatcgccccacttgatt), human LAMP1 (f-cgtacctttccaacagcagc, r-cgctcacgttgtacttgtcc), human MCOLN1 (f-gagtgggtgcgacaagtttc, r-tgttctcttcccggaatgtc), and human Beclin 1 (f- ggtgtctctcgcagattcatc, r-tcagtcttcggctgaggttct). The levels of mRNA expression data were normalized to GAPDH gene expression.

### Western blot

Lysates of harvested cells and midbrain tissues were prepared using RIPA buffer (Thermo Scientific, Waltham, MA, USA) containing phosphatase and protease inhibitors (Sigma-Aldrich). Total protein concentration of the lysates was measured by a BCA protein assay kit (Pierce Biotechnology, Rockford, IL, USA), and then lysates were boiled in 2X Laemmli sample buffer (Bio-Rad, Hercules, CA, USA) containing β-mercaptoethanol. Proteins were resolved by SDS-PAGE and transferred onto polyvinylidene difluoride (PVDF) membranes (Millipore, Burlington, MA, USA). The membrane was incubated overnight at 4 °C with primary antibody against TH (1:1000, Cell Signaling, Danvers, MA, USA), α-synuclein (1:1000, Cell Signaling), p-GSK-3β (1ː1000, Cell Signaling), GSK-3β (1ː1000, Cell Signaling), TFEB (1:1000, Bethyl Laboratories, Montgomery, TX, USA), PARP (1:2000, Cell Signaling), LC3B (1:2000, Novus Biologicals, CO, USA), p62 (1:10000), LAMP1 (1:1000, Abcam, Cambridge, MA, USA), APP (1:2000, Sigma-Aldrich), p-GS (1ː1000, Cell Signaling), GS (1ː1000, Cell Signaling), p-PERK (1:1000, Signalway Antibody, Baltimore, MD, USA), PERK (1ː1000, Cell Signaling), p-eIF2-α (1ː1000, Cell Signaling), eIF2-α (1ː1000, Cell Signaling), Beclin 1 (1:1000, BD bioscience, San Jose, CA, USA), p-MLKL (1ː1000, Abcam, Cambridge, MA, USA), MLKL (1ː1000, GeneTex, Irvine, CA, USA), PP2BAα (1:1000, Santa Cruz), α-tubulin (1:2000, Cell Signaling), and β-actin (1:2500, Thermo Scientific). Then, membranes were incubated with horseradish peroxidase-conjugated secondary antibodies at room temperature for 30 min. Immunoreactivity was detected using the ECL detection system (Pierce Biotechnology), and chemiluminescence signal was read with an Azure Biosystems C300 (Azure Biosystems, Dublin, CA).

### Immunofluorescence

To detect TH^+^ neurons, colocalization of LC3B with α-syn, and necroptosis proteins in midbrain, fixed whole brain samples were sliced into 30 μm tissue sections including *substantia nigra pars compacta* (SNc) and striatum (STR), and sliced samples were blocked with 4% BSA and 0.1% Triton X-100 in PBS for 30 min. The samples were incubated with anti-TH (1:200, Cell Signaling), LC3B (1:200, Cell Signaling), p-MLKL (1:200, Cell Signaling), and α-syn (1:200, Cell Signaling) primary antibody overnight at 4 °C. To analyze colocalization of p-MLKL with Beclin 1 in SH-SY5Y cells, after treatment, the samples were fixed, blocked, and then incubated with anti-Beclin 1 (1:200, BD Biosciences) and p-MLKL (1:200, Abcam). After incubation of primary antibody, the samples were incubated with Alexa Fluor 488 and Alexa Fluor 594-conjugated secondary antibody (Invitrogen) for 2 h at room temperature. Fluorescent images of the samples were obtained using a confocal microscope (Olympus, Tokyo, Japan).

### Fluorescence cell imaging

For imaging of TFEB fluorescence, SH-SY5Y cells were seeded on a 4-well Lab-Tek chamber (Thermo Scientific) and transfected with pEGFP-N1-TFEB plasmid. To detect TFEB nuclear import, transfected cells were treated with 20 μM CORM-A1 for 6 h and 5 μM Torin1 for 3 h. To detect TFEB nuclear export, cells were starved using HBSS for 2 h and then refed complete DMEM for 2 h in the presence or absence of 20 μM CORM-A1 or 20 nM LMB. The cells were fixed in 10% neutral-buffered formalin and then stained with 1 μg/ml DAPI for 20 min. Images of TFEB-GFP location were obtained by an Olympus FV1200 confocal microscope (Olympus).

### MLKL oligomerization

Reducing samples were prepared by lysing cells with 2X Laemmli sample buffer (Bio-Rad) with β-mercaptoethanol. For non-reducing SDS-PAGE, samples were prepared with 2X Laemmli sample buffer (Bio-Rad) without β-mercaptoethanol, and then samples were directly loaded into the acrylamide gel.

### Phosphatase assay

Human MLKL overexpressed in SH-SY5Y cells was treated with MPP^+^ or TBZ and then immunoprecipitated with anti-MLKL antibody. As described previously [63], MLKL immunoprecipitates from SH-SY5Y cells were resuspended in assay buffer (20 mM Tris, 10 mM MgCl_2_, 0.1 mM CaCl2, and 1 mg/ml bovine serum albumin, pH 7.5) and incubated with or without 500 units recombinant human calcineurin (Enzo Life Sciences, Farmingdale, NY, USA) for 1 h at 30 °C. MLKL dephosphorylation was determined by immunoblotting.

### Subcellular fractionation

Nuclear and cytoplasmic fractions were extracted from SH-SY5Y cells using a nuclear/cytosol fractionation kit (Biovision, Mountain View, CA, USA) according to the manufacturer’s instructions. Briefly, harvested cells were resuspended with cytosolic extraction buffer A (CEB-A) and incubated for 10 min on ice prior to addition of CEB-B. After 1 min, the lysates were centrifuged at 4 °C for 5 min at 16,000 × *g* in a microcentrifuge, and the supernatants were kept as the cytoplasmic fractions. The nuclear pellet was resuspended with nuclear extraction buffer (NEB). Suspensions were centrifuged at 4 °C for 10 min at 16,000 × *g*, and the supernatant was kept as a nuclear fraction. The cytoplasmic and nuclear fractions were subjected to Western blotting. Anti-PARP and anti-α-tubulin antibodies were used to assess the purity of nuclear and cytoplasmic fractions, respectively.

### Sytox green staining

SH-SY5Y cells were pretreated with 20 μM CORM-A1 for 2 h and then treated with 1 mM MPP^+^ for 24 h. The cells were stained with 125 nM Sytox Green (Invitrogen) for 15 min. Dead cells were then directly detected by using flow cytometry with a FACSCanto flow cytometry system (BD Biosciences), and data were analyzed by FlowJo V10 software (TreeStar, Ashland, OR, USA).

### LDH cytotoxicity assay

To measure necrotic cell death, the LDH released from cells into culture supernatants was measured using the LDH cytotoxicity assay kit (Biovision, Mountain View, CA, USA) according to manufacturer’s protocol. Briefly, culture supernatant and cells were harvested separately. The supernatants were dispensed into 96-well plates, and LDH reagents were added. To quantify LDH activity, the optical density of samples was read at 490 nm on a SpectraMax iD3 (Molecular Devices, Sunnyvale, CA, USA).

### Statistical analysis

All data were expressed as mean ± SD. Differences between the corresponding control and the experimental groups were analyzed using by one-way ANOVA with Tukey *post hoc* test. Data of siRNA-transfected cells were assessed by two-way ANOVA with Bonferroni post-tests. Data were analyzed and presented with GraphPad Prism software (San Diego, CA, USA).

## Supplementary information

Supplementary Information

uncropped western blotting

## Data Availability

The data that supports the findings of this study are available in the manuscript and supplementary material of this article.
