# Supplementary material for: CO confers neuroprotection via activating the PERK-calcineurin pathway and inhibiting necroptosis
Source: Cell Death Discov. 2025 May 27;11:254. doi: 10.1038/s41420-025-02530-9 (PMC12116729; doi:10.1038/s41420-025-02530-9)
Supplement: Supplementary file 1 — Supplementary Information [file 41420_2025_2530_MOESM1_ESM.docx]

**Supplementary Information**

**CO Confers Neuroprotection via Activating the PERK-Calcineurin Pathway and Inhibiting Necroptosis**

Jeongmin Park ^1, *^, LiHua Jin^2, *^, Hyun-Chul Song^3^, Yingqing Chen^4^, Eun Young Jang^5^, Gyu Hwan Park^6^, Chae Ha Yang^7^, Stefan W. Ryter^8^, Jeong Woo Park^3^, Min Zheng^9^, Yeonsoo Joe^1*^, and Hun Taeg Chung^1*^

^1^College of Korean Medicine, Daegu Haany University, Gyeongsan 38610, Republic of Korea

^2^School of Nursing, YanBian University, Yanji, 133000, China, ^3^School of Biological Sciences, University of Ulsan, Ulsan, 44610, Korea, ^4^Department of Pharmacology, Dalian University Medical College, Dalian, 116622, China, ^5^Department of Advanced Toxicology Research, Korea Institute of Toxicology, Daejeon, 34114, Korea, ^6^College of Pharmacy, Research Institute of Pharmaceutical Sciences, Kyungpook National University, Daegu, 41566, Korea, ^7^College of Korean Medicine, Daegu Haany University, Daegu, 42158, Korea, ^8^Proterris Inc., Boston, MA, USA, 02118, USA, ^9^Department of Neurology, Affiliated Hospital of YanBian University, Yanji, 133000, China

***Address co-correspondence to:**

**Yeonsoo Joe,** [**joeyeonsoo@dhu.ac.kr**](mailto:joeyeonsoo@dhu.ac.kr)**;**

**Hun Taeg Chung, chunght@dhu.ac.kr**

**Yeonsoo Joe Hun Taeg Chung**

College of Korean Medicine, College of Korea Medicine,

Daegu Haany University, Gyeongsan, Korea Daegu Haany University, Gyeongsan, Korea

Tel: +82-53-819-1868 Tel: +82-53-819-1868

E-mail :joeyeonsoo@dhu.ac.kr E-mail : chunght@dhu.ac.kr


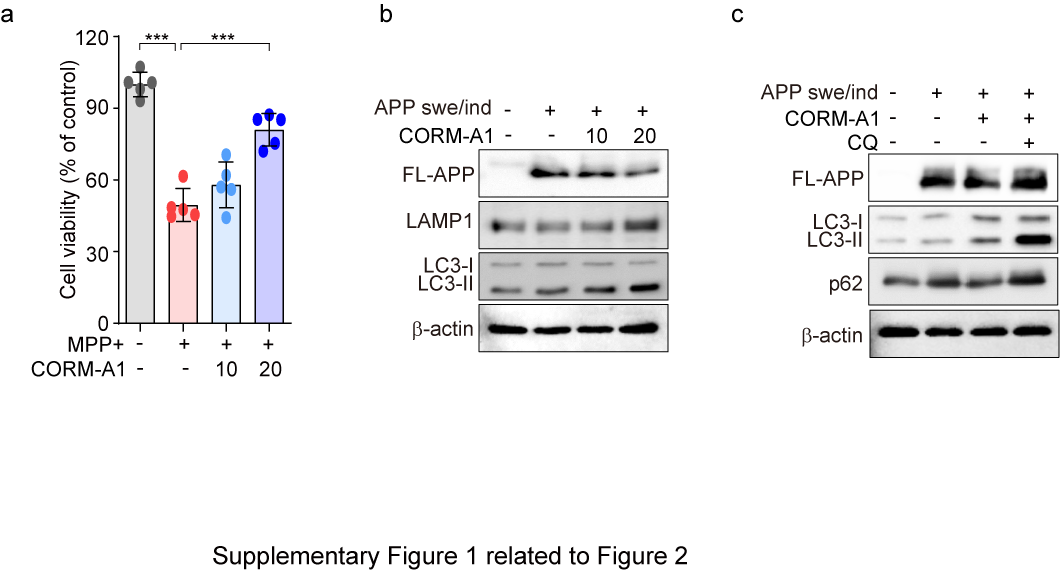


**Supplementary Fig. 1 (related to Fig. 2)**

**a** SH-SY5Y cells were treated with CORM-A1 at the indicated concentrations (10 and 20 μM) for 1 h and then treated with 1 mM MPP^+^ for 24 h. Cell viability was measured by the WST-8 assay. **b** SH-SY5Y cells were transfected with pCAX-APP-Swe/Ind and then treated with 10 or 20 μM CORM-A1 for 12 h. After 12 h, FLAPP, LAMP1, and LC3-I/-II expression in treated cells was determined by western immunoblotting. **c** SH-SY5Y cells were transfected with pCAX-APP-Swe/Ind for 36 h. The cells were treated with 20 μM CORM-A1 for 12 h in the presence or absence of the autophagy inhibitor CQ (10 μM). Protein expressions of FL-APP, LAMP1, and p62 were detected by western blotting. Data were expressed as mean ± SD; ****p*<0.001.


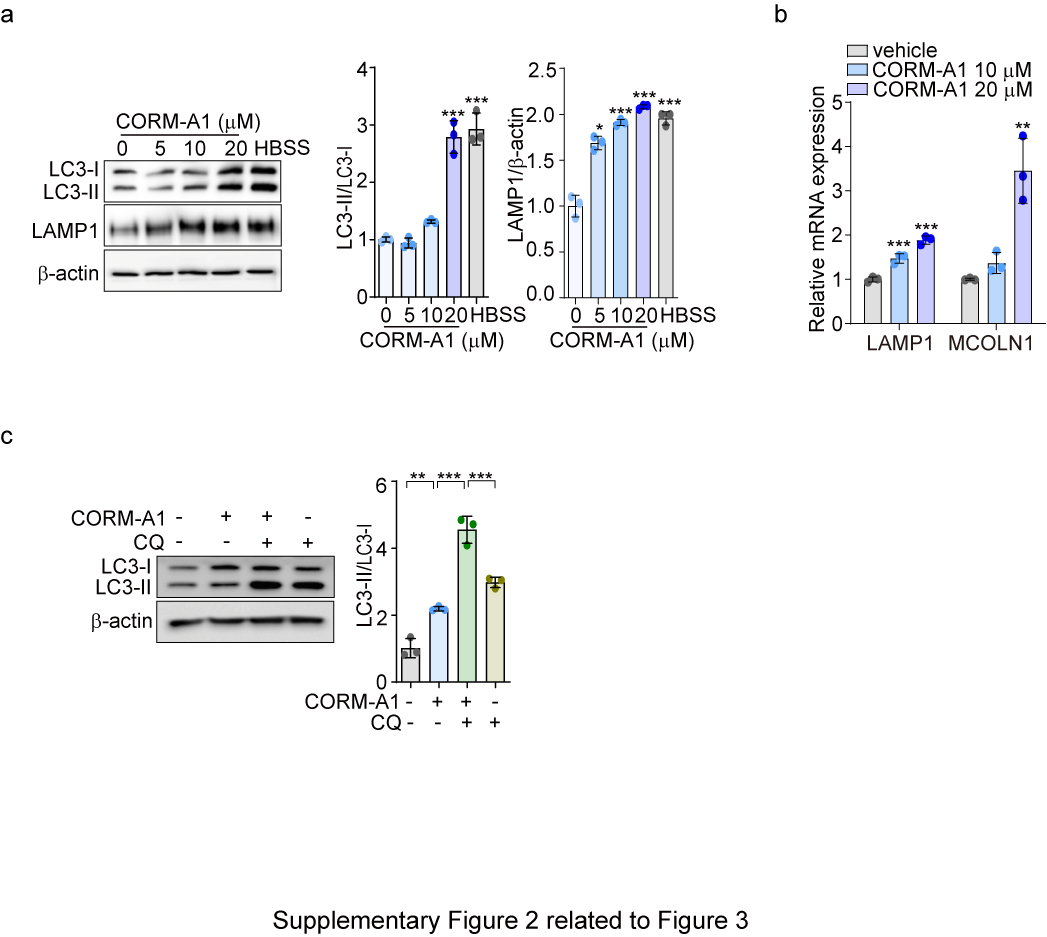


**Supplementary Fig. 2 (related to Fig. 3)**

**a** SH-SY5Y cells were treated with CORM-A1 at the indicated concentrations (5, 10, and 20 μM) for 12 h. The levels of LC3-I/-II and LAMP1 expression were assessed by western blotting. **b** Cells were treated with 10 and 20 μM CORM-A1 for 12 h. To measure lysosomal genes, treated cells were analyzed for LAMP1 and MCOLN1 mRNA using qRT-PCR. **c** SH-SY5Y cells were pretreated with 10 μM CQ for 1 h followed by treatment with 20 μM CORM-A1 for 12 h. LC3-II conversion was determined by western blotting. Data were expressed as mean ± SD; **p*<0.05, ***p*<0.01, and ****p*<0.001.


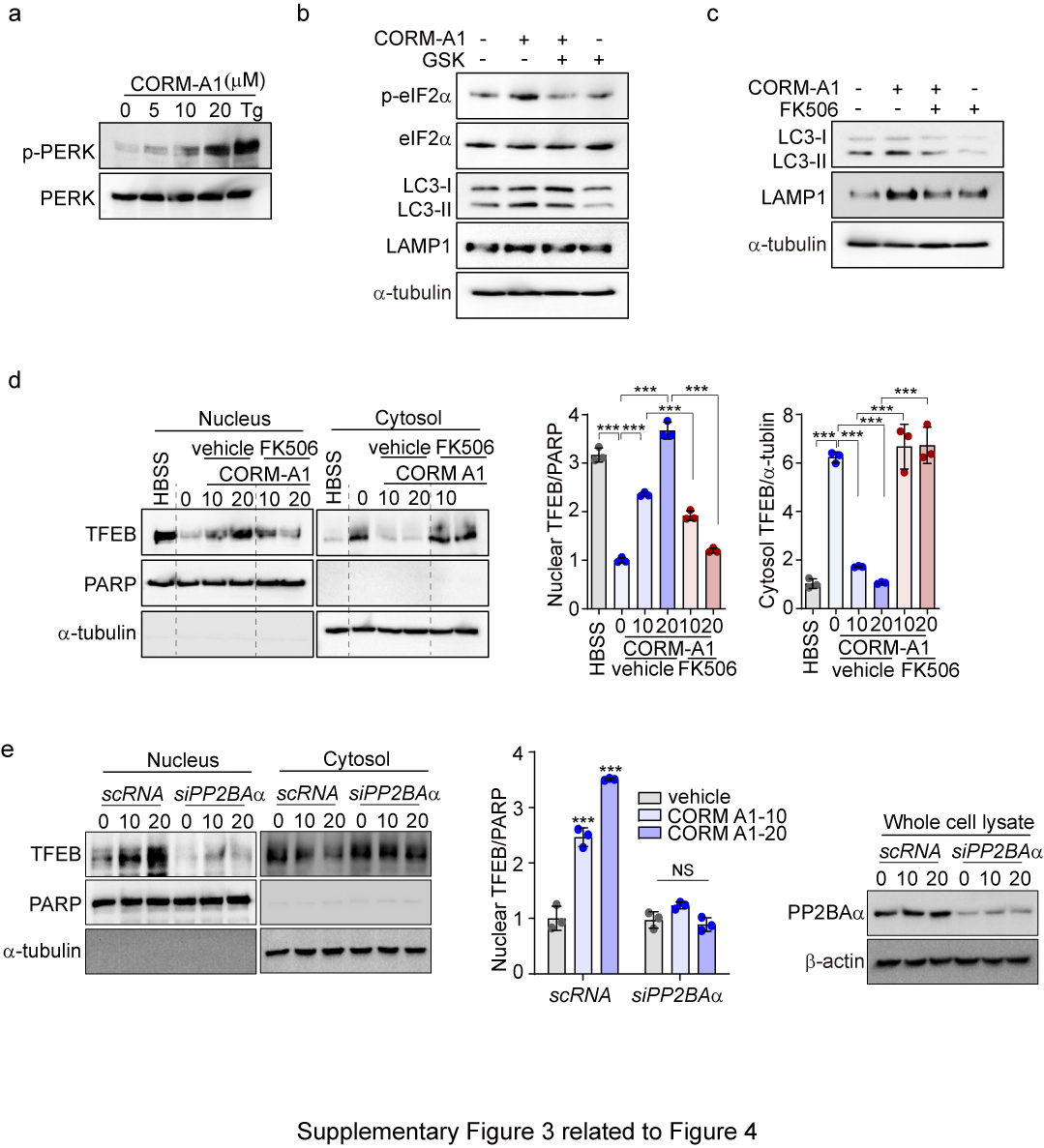


**Supplementary Fig. 3 (related to Fig. 4)**

**a** SH-SY5Y cells were treated with CORM-A1 at various concentration (5, 10, and 20 μM) for 6 h. 1 μM Tg treatment was used as a positive control. The protein expression of p-PERK and PERK was determined by western blotting. **b** SH-SY5Y cells were pretreated with 1 μM GSK2606414 for 30 min and then with 20 μM CORM-A1 for 6 h. The protein expression of p-eIF2α, eIF2α, LC3-I/-II, and LAMP1 was detected by western blotting. **c** SH-SY5Y cells were treated with the calcineurin inhibitor FK506 (10 μM) for 30 min followed by treatment with 20 μM CORM-A1 for 12 h. The protein levels of LC3-I/-II and LAMP1 were detected by western blotting. **d** Cells were treated with 10 and 20 μM CORM-A1 for 6 h in the presence or the absence of 10 μM FK506. SH-SY5Y cells incubated with HBSS medium were used as a positive control. Nuclear and cytoplasmic fractions extracted from SH-SY5Y cells were analyzed by western blotting using TFEB antibody. **e** SH-SY5Y cells transfected with *scRNA* or *siPP2BAα* were treated with 10 and 20 μM CORM-A1 for 6 h. TFEB nuclear translocation was analyzed by western blotting in nuclear and cytoplasmic fraction extracts (*left panel*). Nuclear translocation of TFEB was quantified (*middle panel*). Knockdown of PP2BAα was analyzed by western blotting in whole cell lysate (*right panel*). Data were expressed as mean ± SD; ****p*<0.001, and NS, not significant.


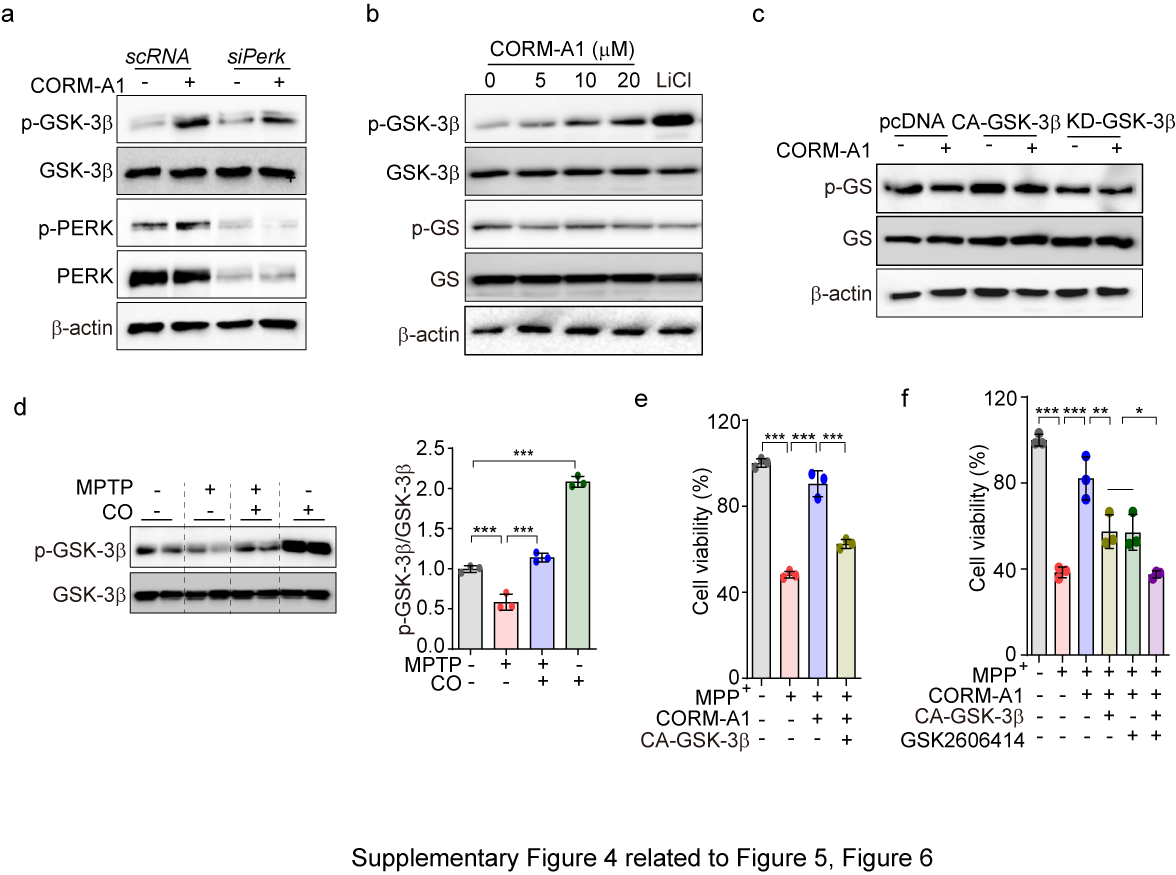


**Supplementary Fig. 4 (related to Fig. 5, Fig. 6)**

**a** SH-SY5Y cells were transfected with scramble RNA (scRNA) or siRNA against PERK (si*Perk*) and then treated with 20 μM CORM-A1 for 2 h. Levels of p-GSK-3β, GSK-3β, p-PERK, and PERK expression were assessed by western blotting. **b** SH-SY5Y cells were treated with CORM-A1 at the indicated concentrations (5, 10, and 20 μM) for 2 h. p-GSK-3β, GSK-3β, p-GS, and GS from treated cells were measured by western blotting. 20 mM LiCl treatment was used as a positive control. **c** SH-SY5Y cells were transfected with pcDNA3.1, CA-GSK-3β, and KD- GSK-3β followed by treatment with 20 μM CORM-A1 for 2 h. Protein expression of p-GS and GS, a downstream target of GSK-3β, was determined by western blotting. **d** 8-week-old WT mice (*n*=5) were pretreated with 250 ppm CO gas (2 h/day) for 13 days. Starting on day 4, mice were injected intraperitoneally with 25 mg/kg MPTP once daily for 7 consecutive days. Inhibitory phosphorylation of GSK-3β (Ser 9) in midbrain was assessed by western blotting (*left panel*). Quantification of phosphorylated GSK-3β is shown in the *right panel*. **e** CA-GSK-3β-transfected SH-SY5Y cells were treated with 20 μM CORM-A1 for 1 h, followed by stimulation with 1mM MPP^+^ for 24 h. Cell viability was determined by WST-8 assay. **f** SH-SY5Y cells were treated with 1 mM MPP^+^ for 24 h in the presence or absence of 20 μM CORM-A1 after the cells were transfected with CA-GSK-3β or treated with 1 μM GSK2606414. Cell viability was measured by WST-8 assay. Data were expressed as mean ± SD; **p*<0.05, ***p*<0.01, ****p*<0.001, and NS, not significant.


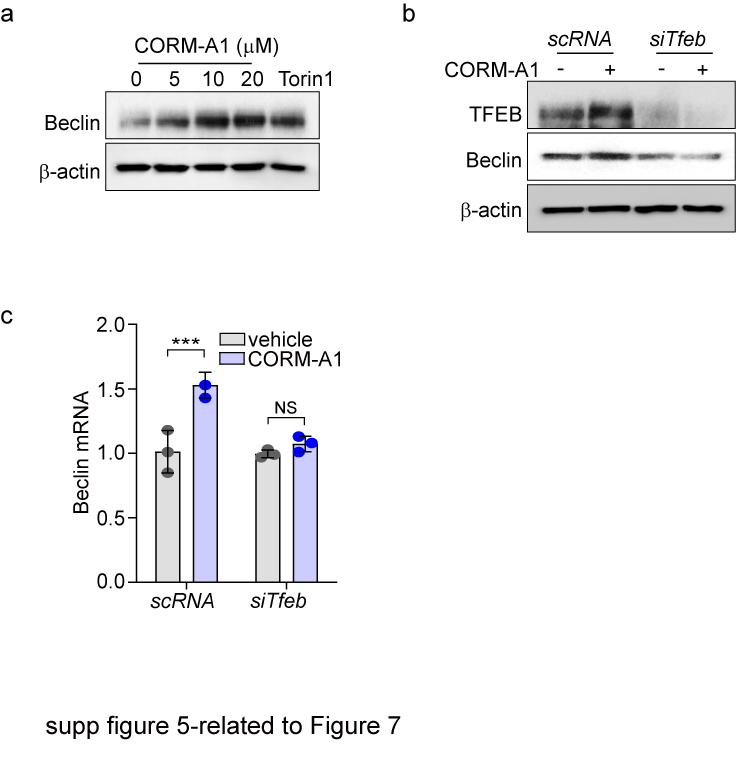


**Supplementary Fig. 5 (related to Fig. 7)**

**a** SH-SY5Y cells were treated with CORM-A1 at the indicated concentrations (5, 10, and 20 μM) for 12 h. 5 μM Torin 1 treatment was used as a positive control. Protein expression of Beclin 1 was detected by western blotting. **b, c** SH-SY5Y cells were transfected with scRNA or si*Tfeb* and then treated with 20 μM CORM-A1 for 12 h. The levels of TFEB and Beclin 1 protein expression were analyzed by western blotting (**b**), and the level of Beclin 1 mRNA was determined by qRT-PCR (**c**). Data were expressed as mean ± SD; ****p*<0.001 and NS, not significant.
