# Supplementary figures and images for: CO confers neuroprotection via activating the PERK-calcineurin pathway and inhibiting necroptosis
Source: Cell Death Discov. 2025 May 27;11:254. doi: 10.1038/s41420-025-02530-9 (PMC12116729; doi:10.1038/s41420-025-02530-9)

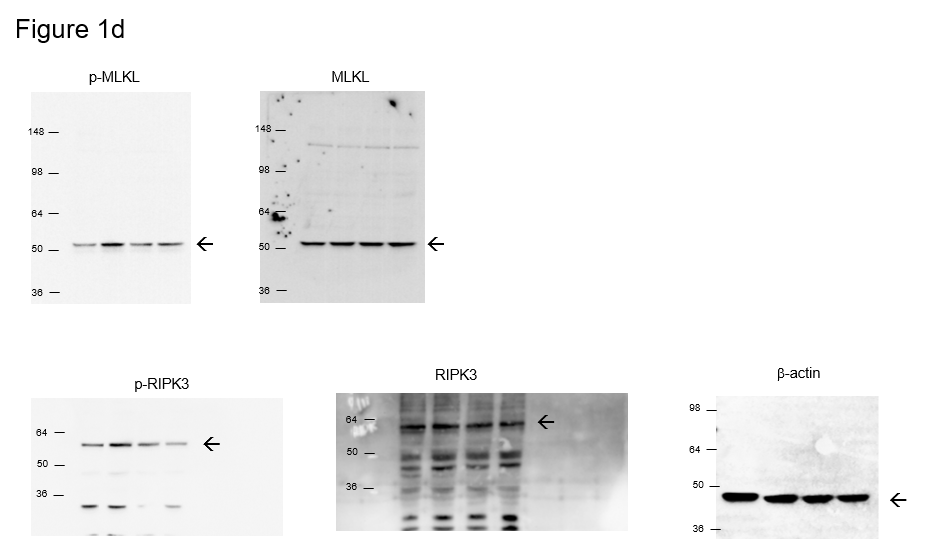


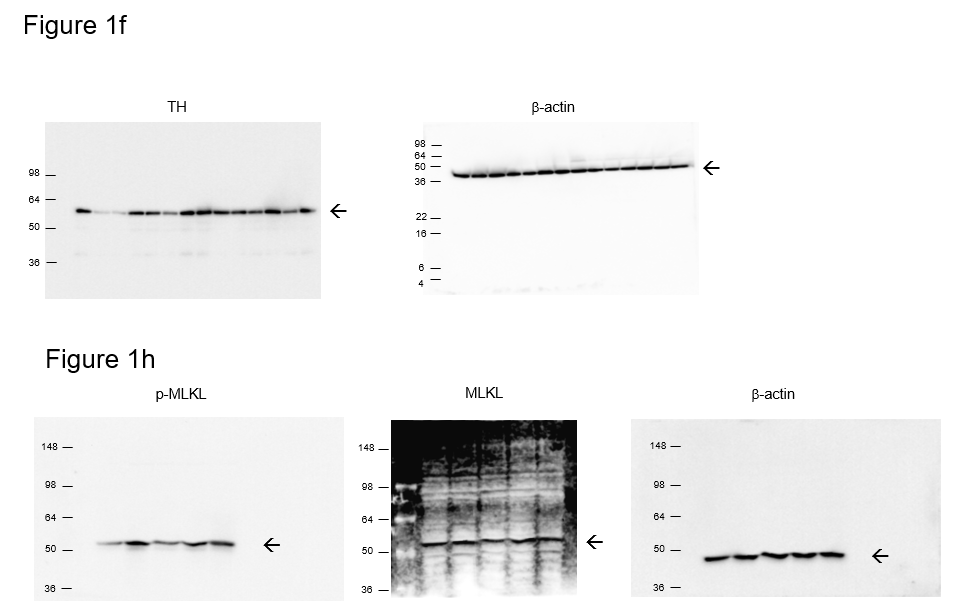


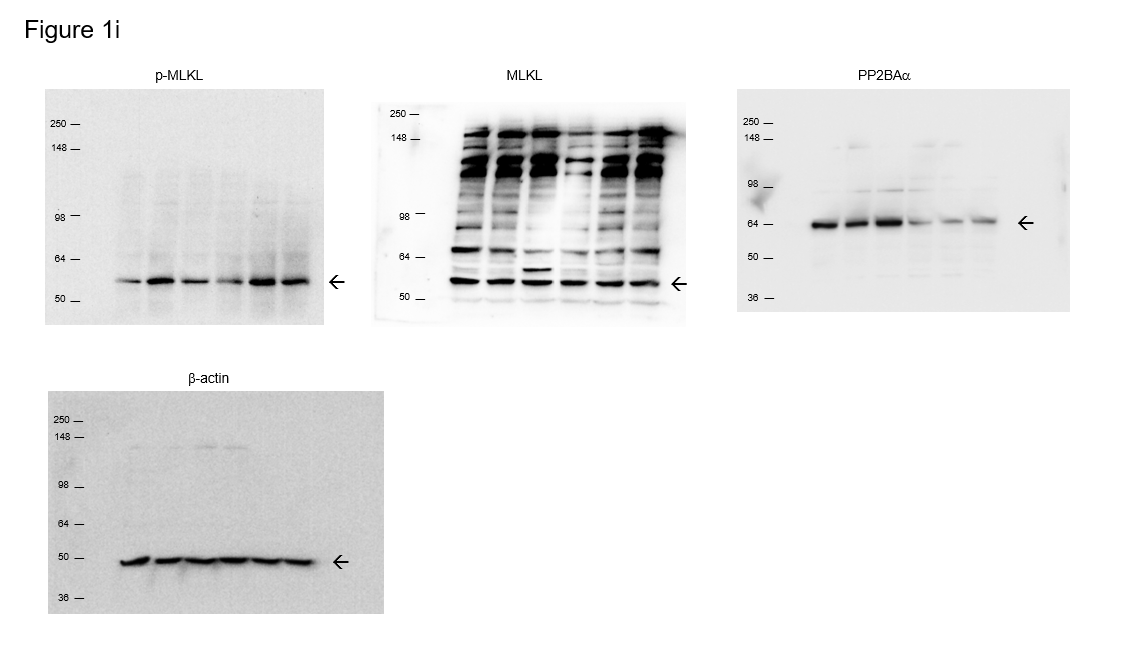


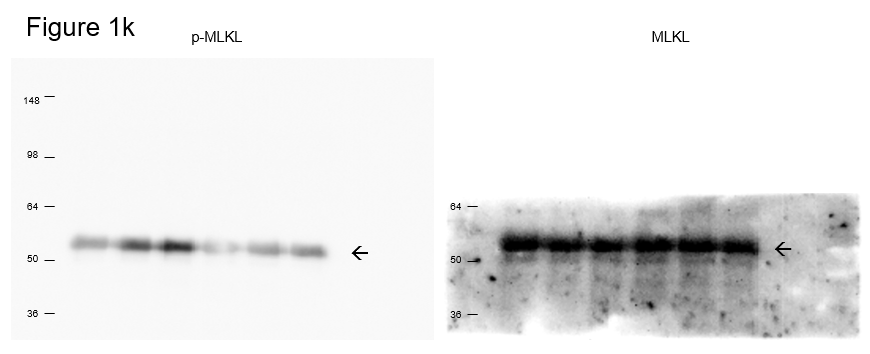


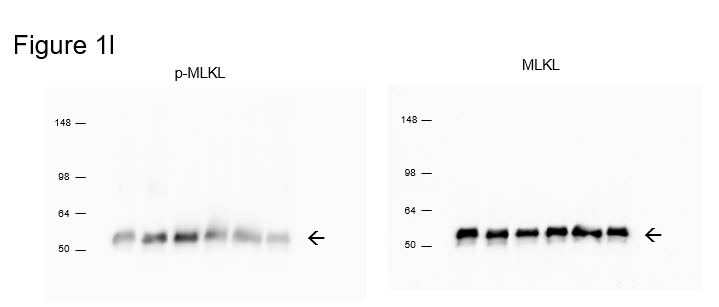


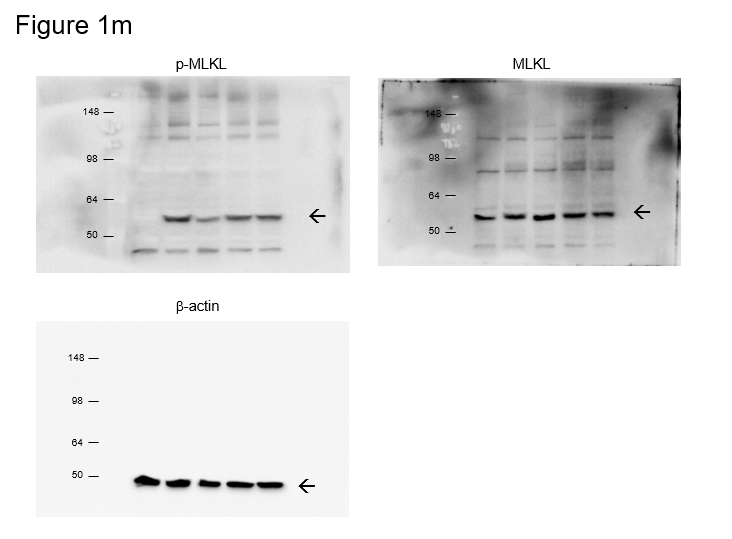


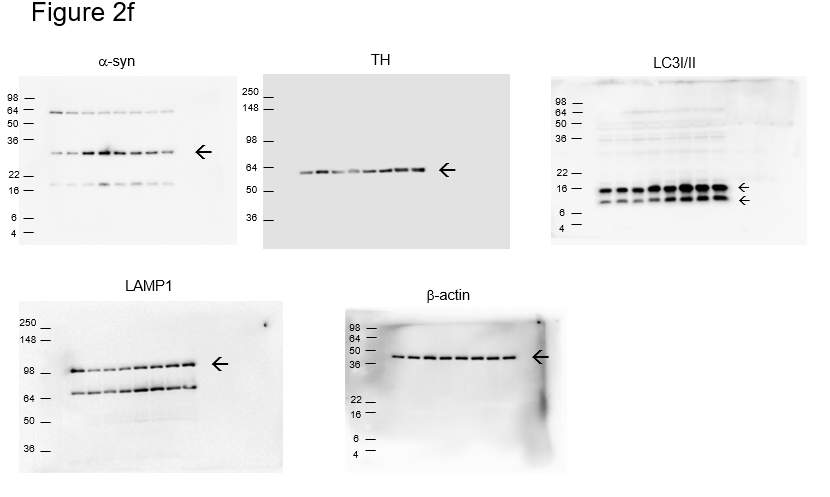


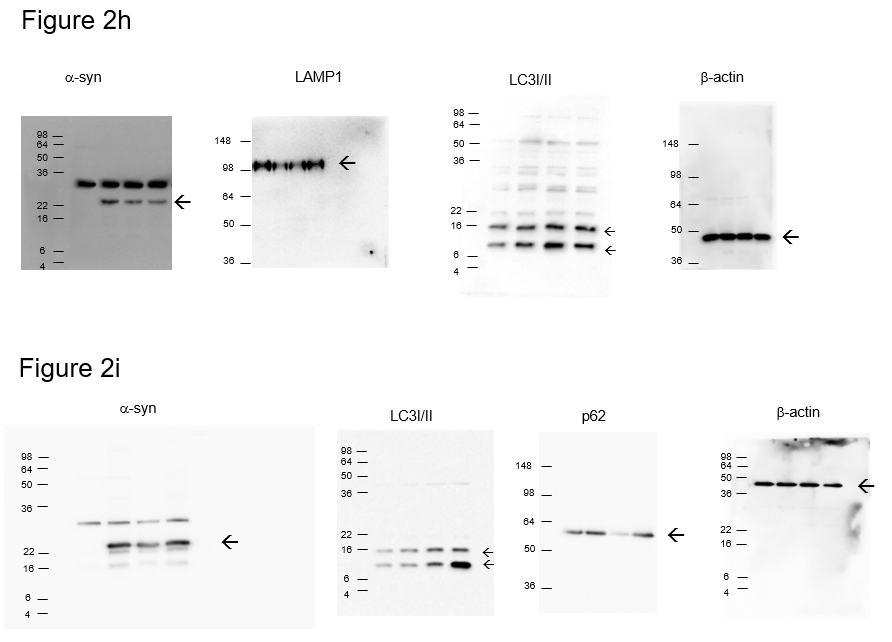


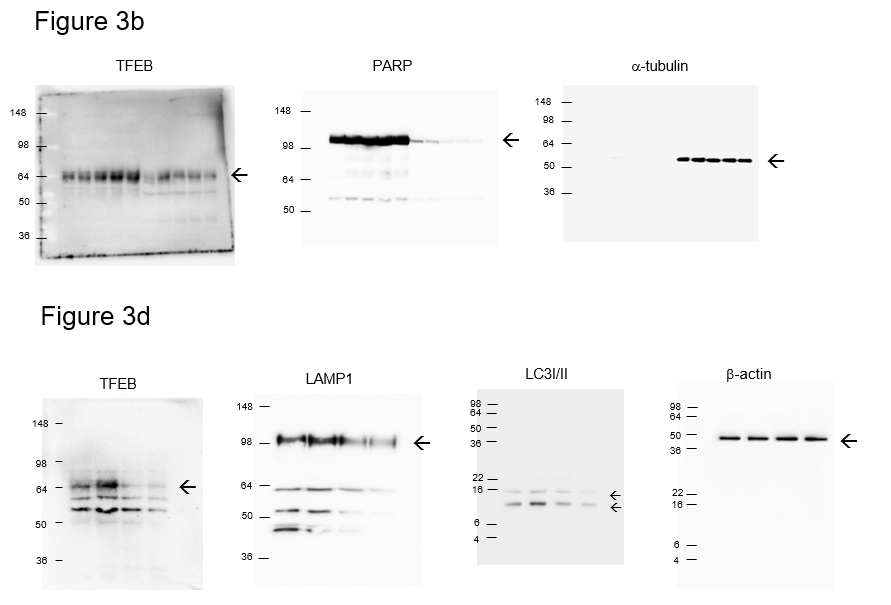


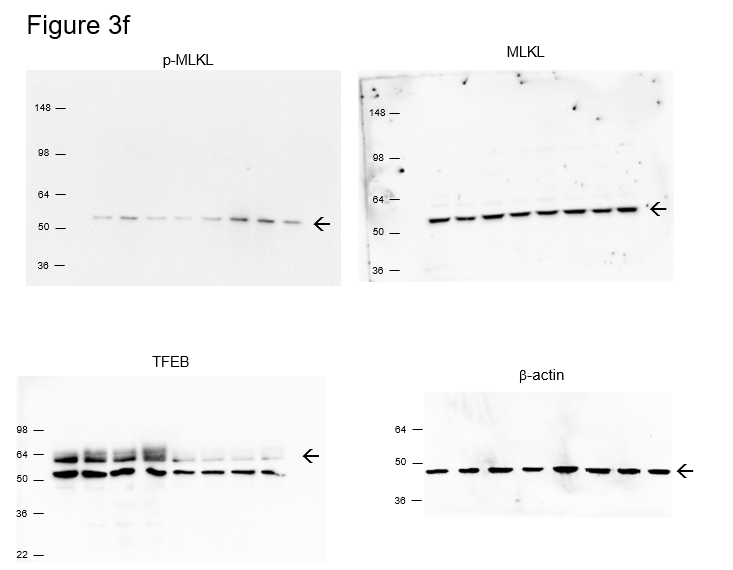


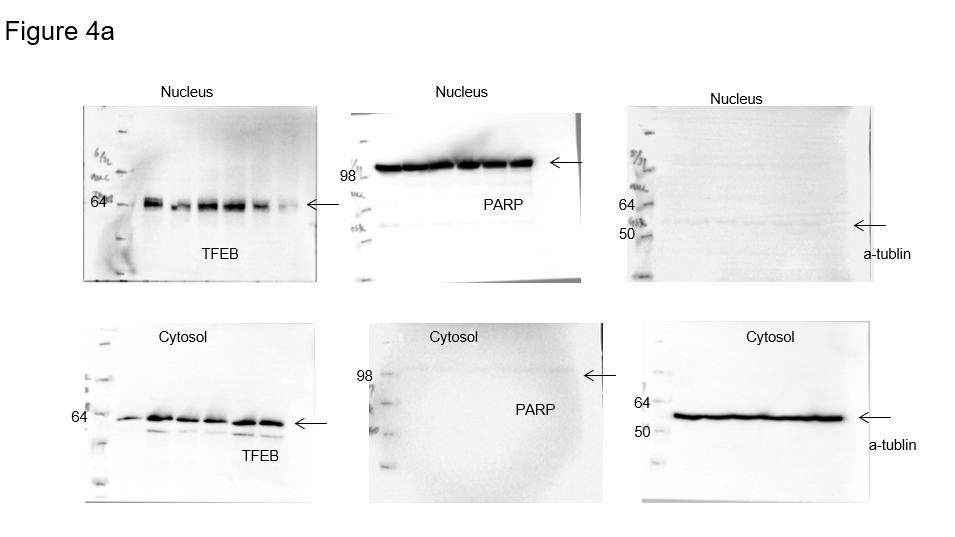


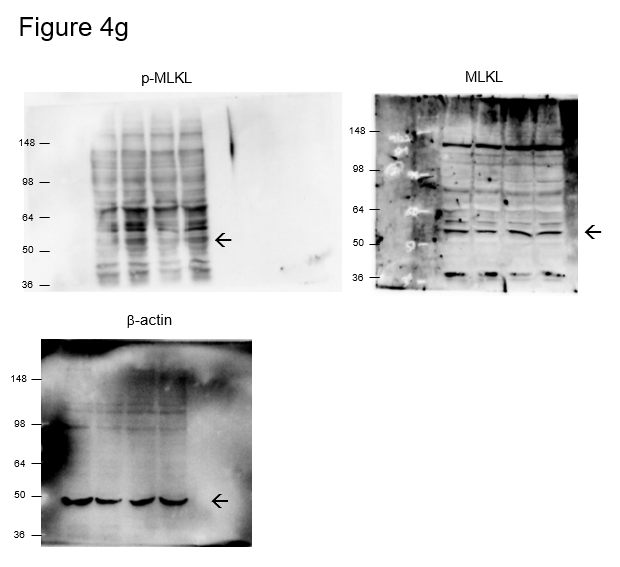


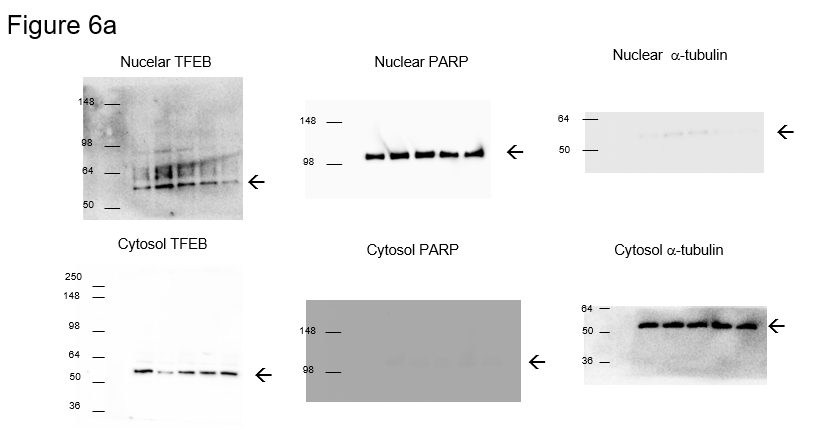


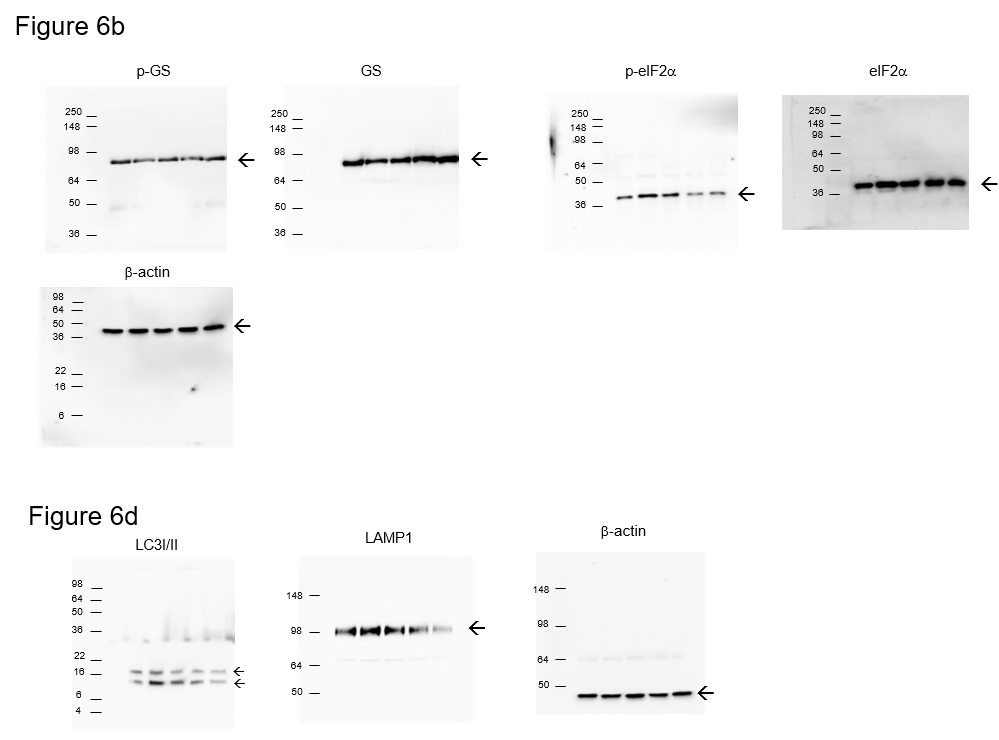


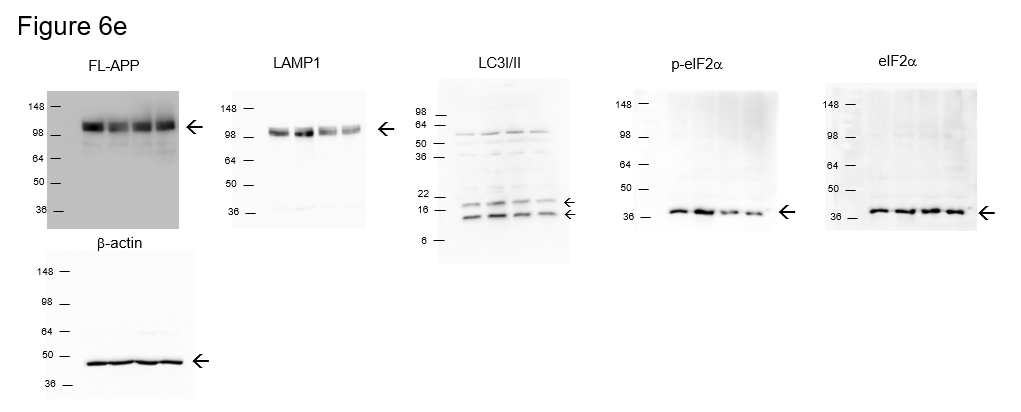


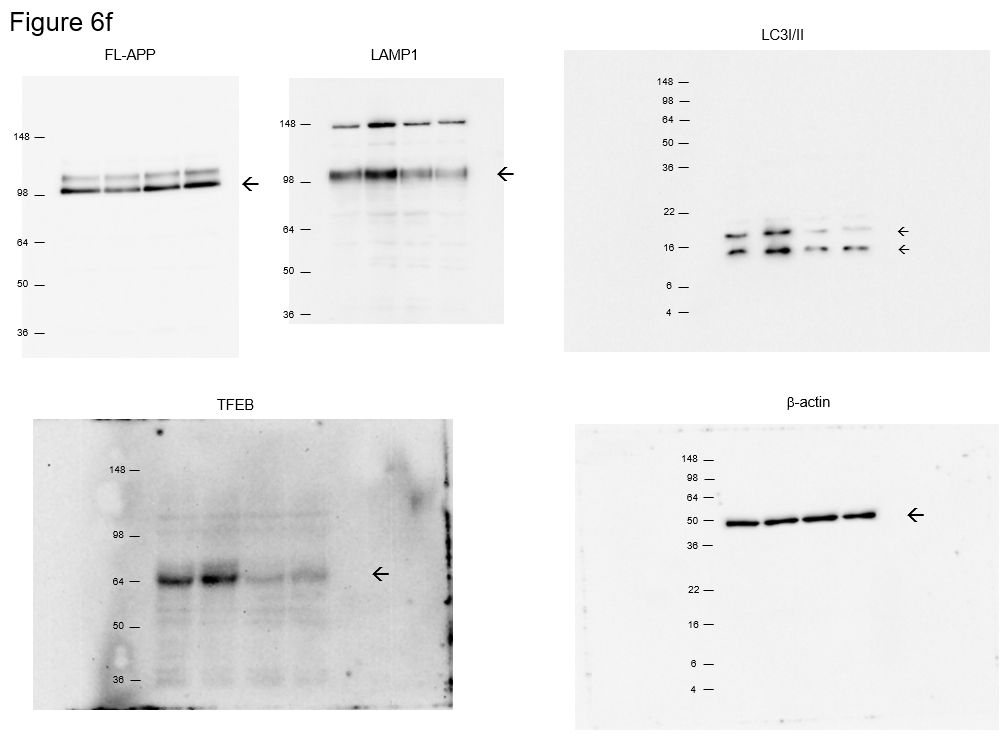


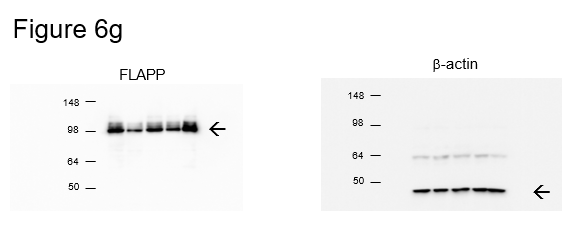


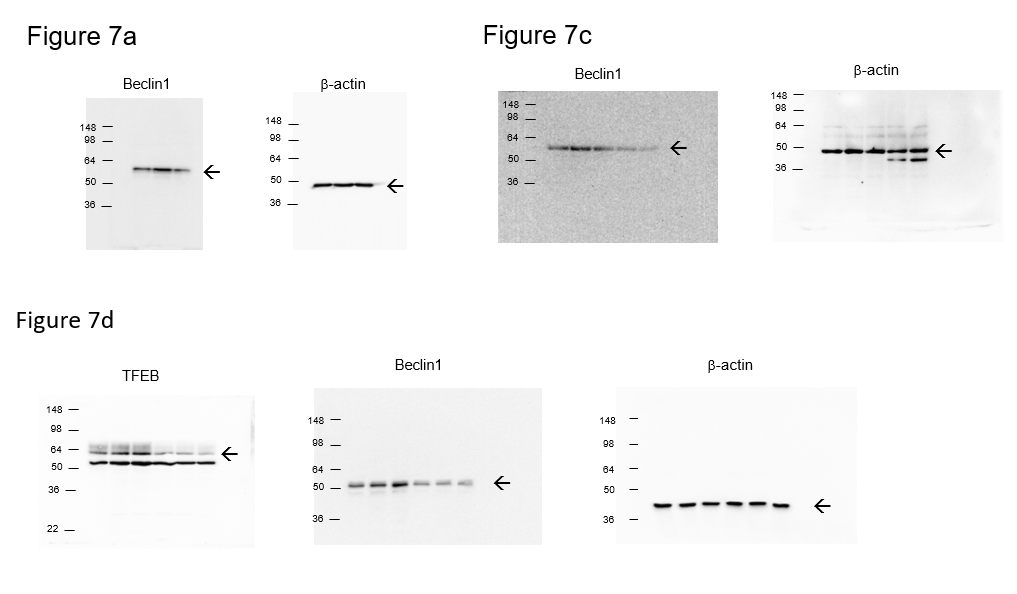


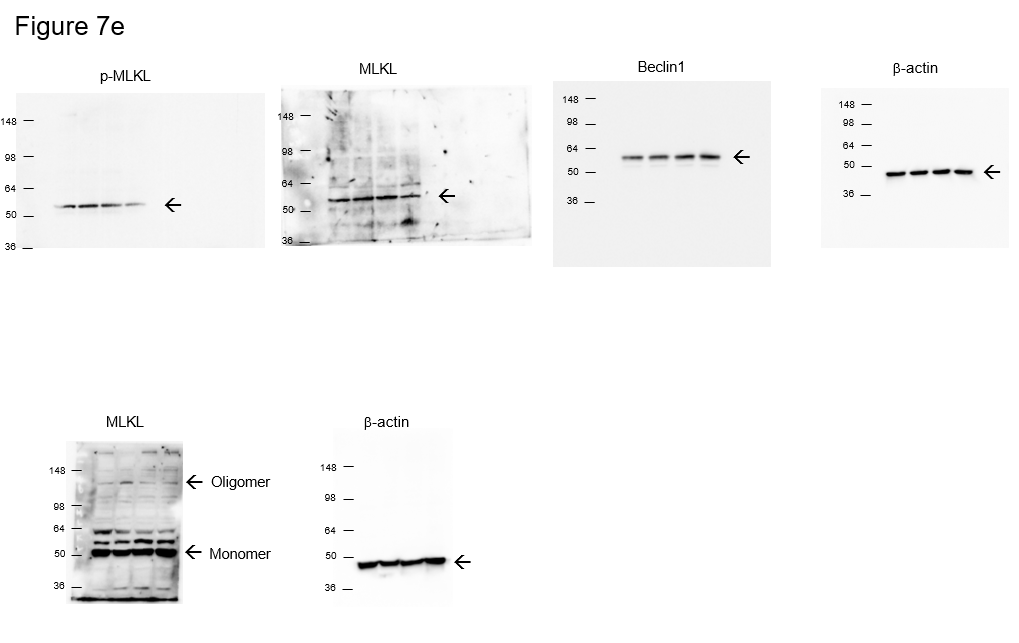


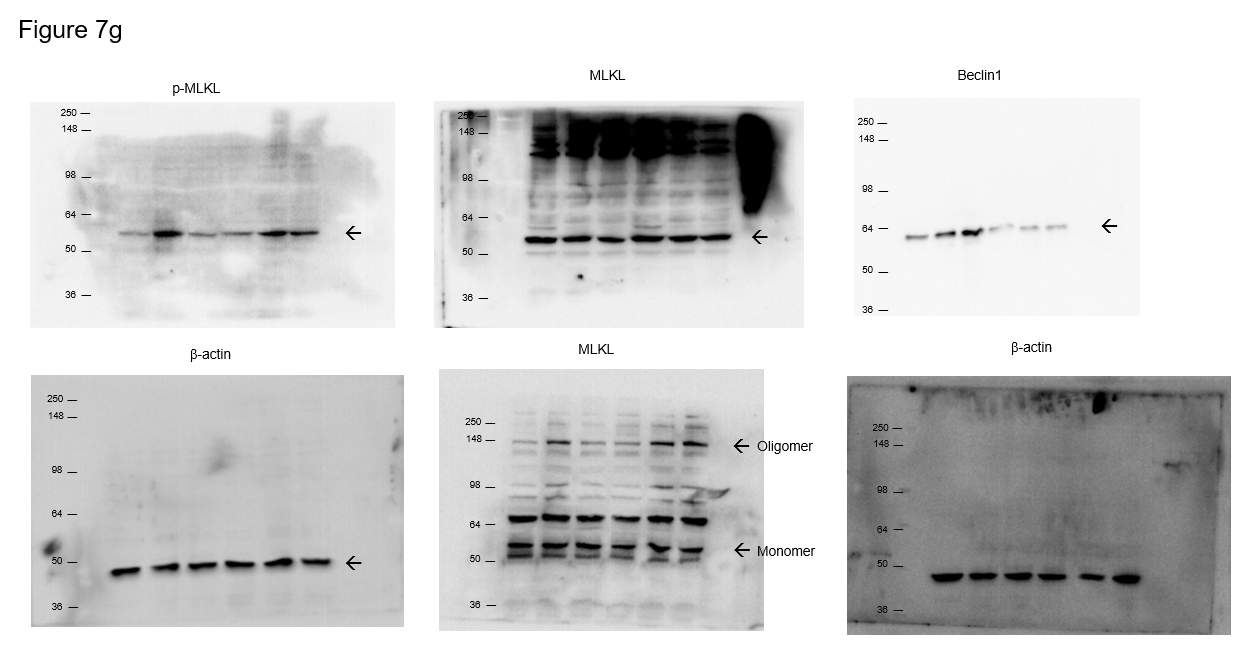


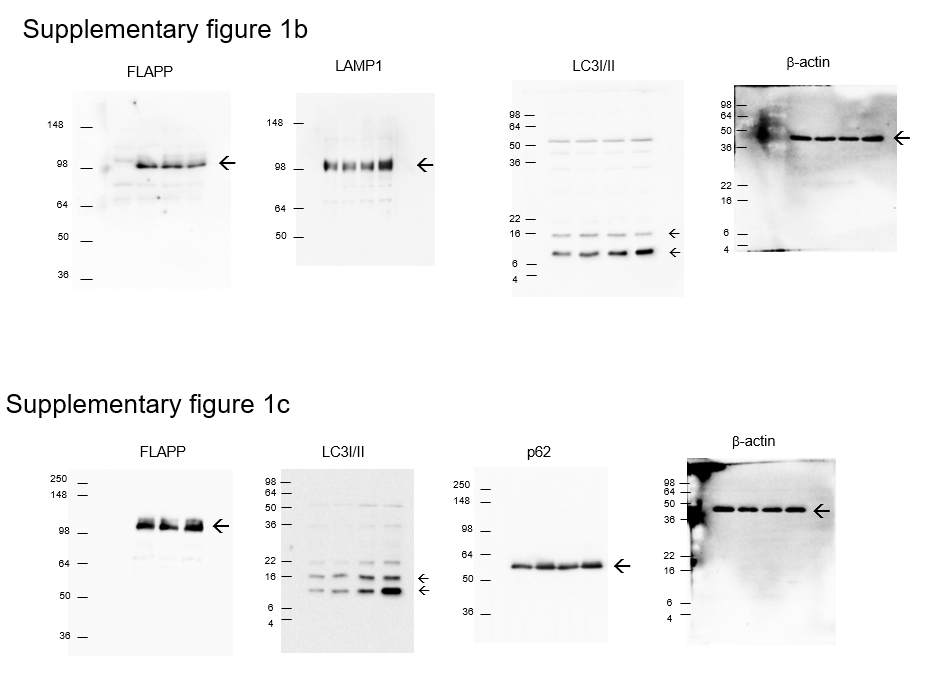


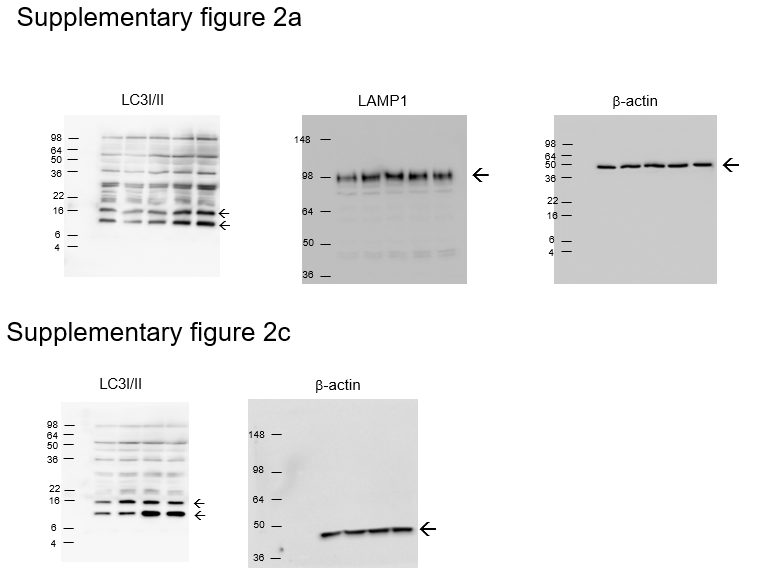


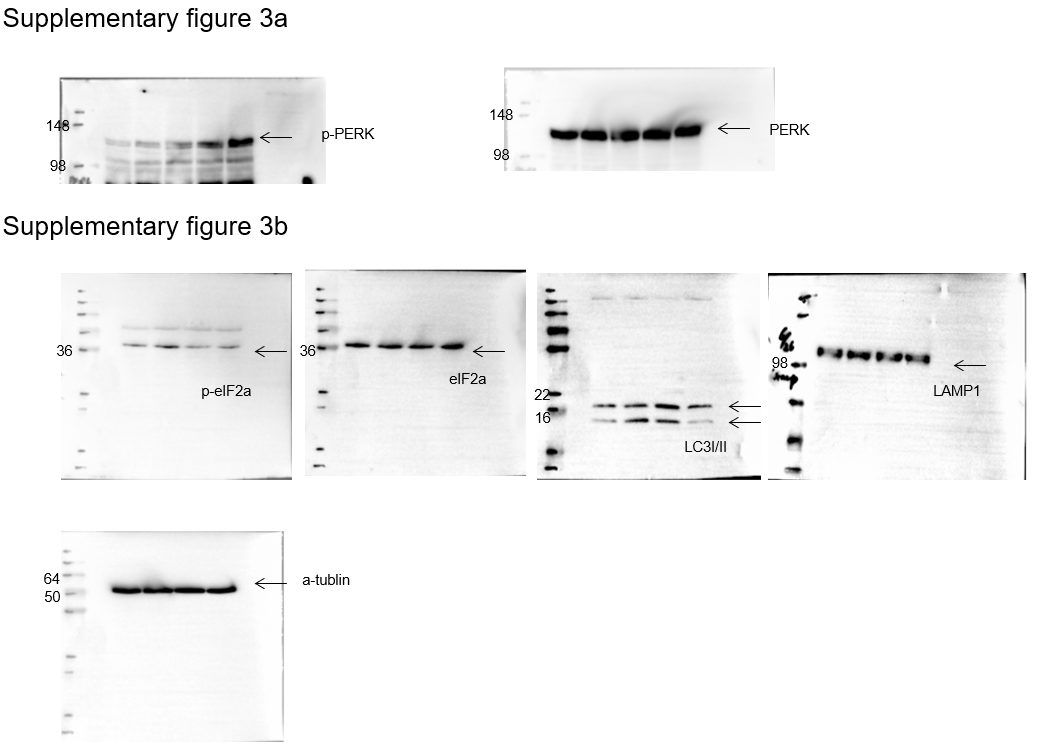


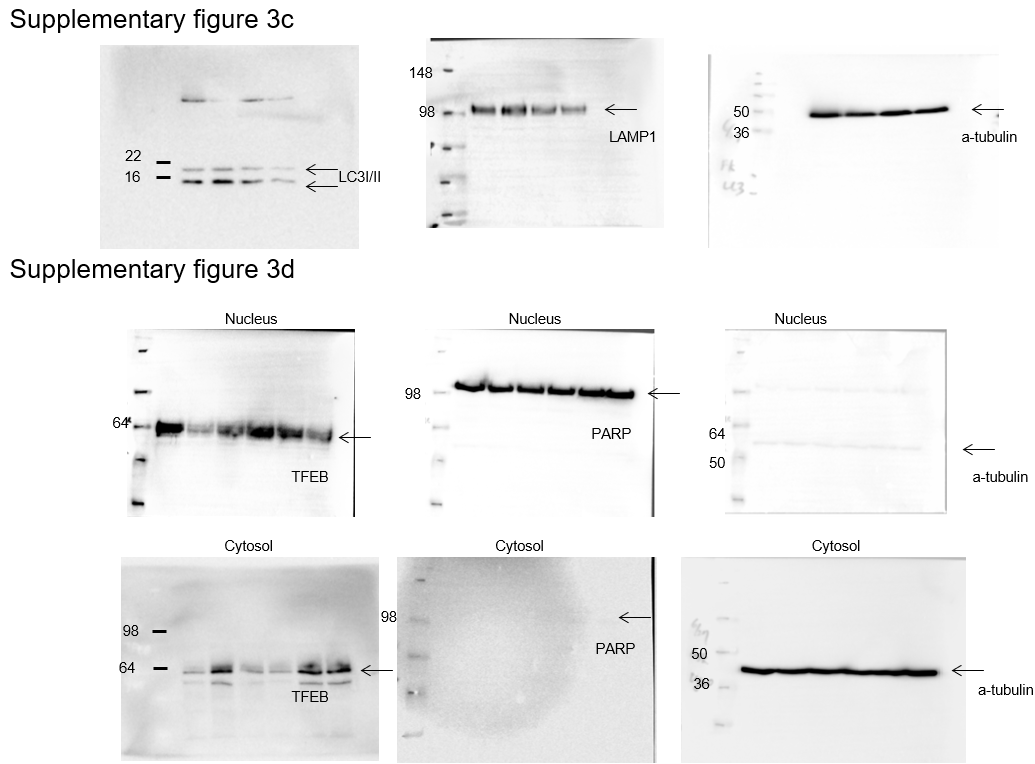


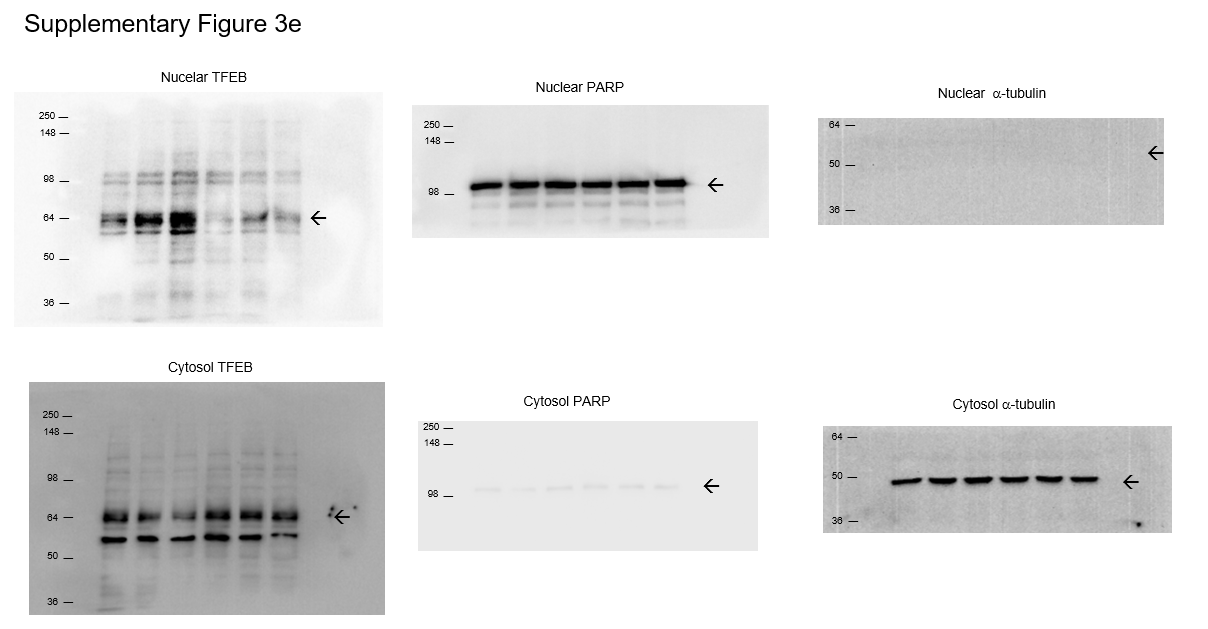

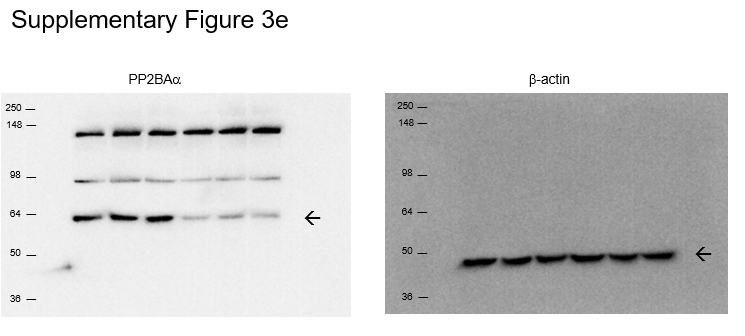


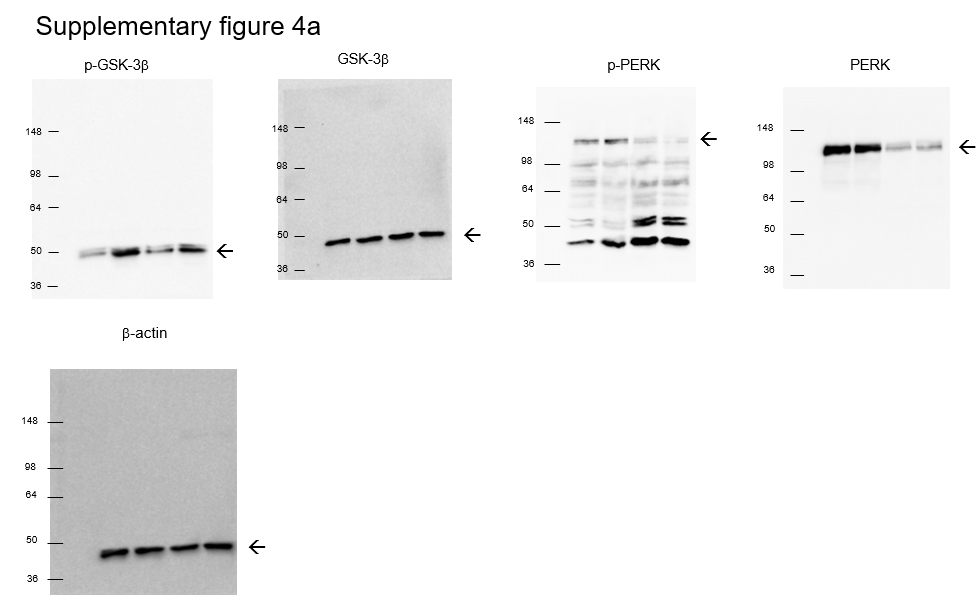

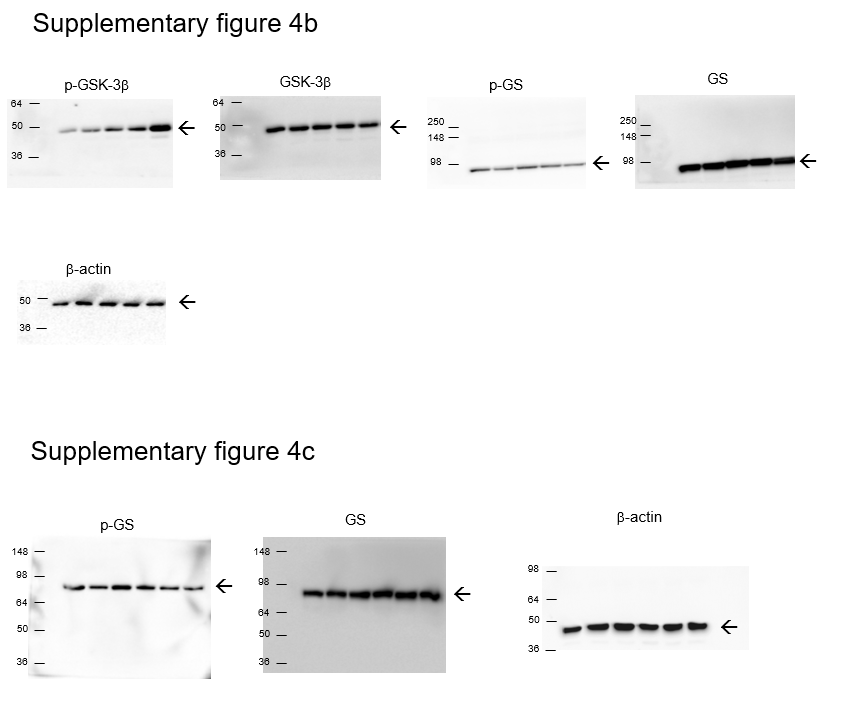


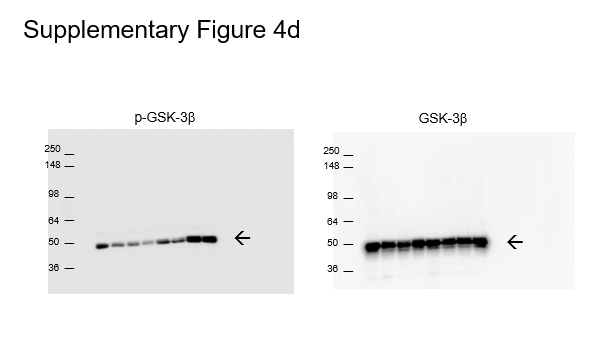


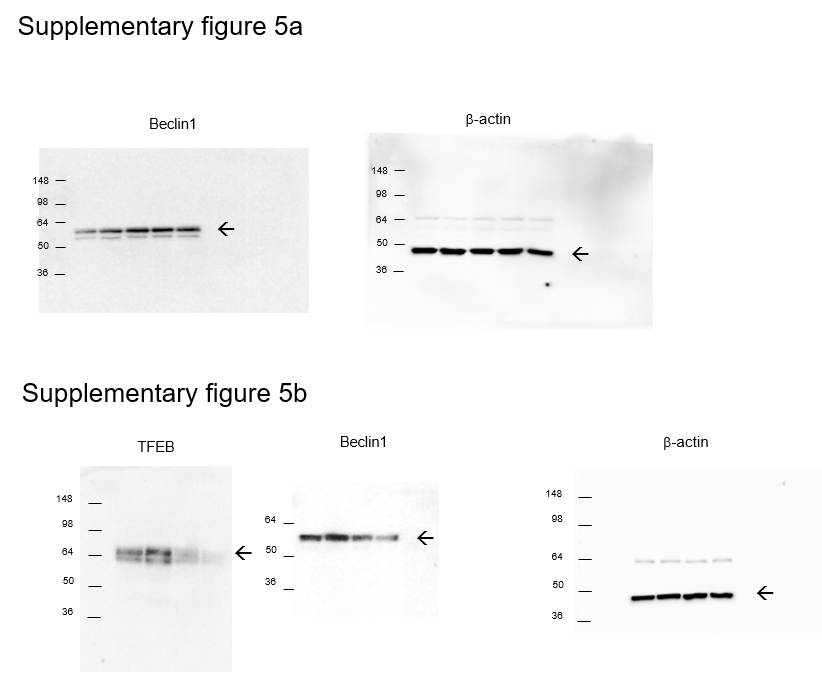

Supplement: Supplementary file 2 — uncropped western blotting [file 41420_2025_2530_MOESM2_ESM.docx]
